# Reconstructing the regulatory circuit of cell fate determination in yeast mating response

**DOI:** 10.1371/journal.pcbi.1005671

**Published:** 2017-07-24

**Authors:** Bin Shao, Haiyu Yuan, Rongfei Zhang, Xuan Wang, Shuwen Zhang, Qi Ouyang, Nan Hao, Chunxiong Luo

**Affiliations:** 1 Center for Quantitative Biology, Academy for Advanced Interdisciplinary Studies, Peking University, Beijing, China; 2 The State Key Laboratory for Artificial Microstructures and Mesoscopic Physics, School of Physics, Peking University, Beijing, China; 3 School of Informatics and Computing, Indiana University, Bloomington, Indiana, United States of America; 4 Peking-Tsinghua Center for Life Sciences, Peking University, Beijing, China; 5 Section of Molecular Biology, Division of Biological Sciences, University of California San Diego, La Jolla, California, United States of America; Rutgers University, UNITED STATES

## Abstract

Massive technological advances enabled high-throughput measurements of proteomic changes in biological processes. However, retrieving biological insights from large-scale protein dynamics data remains a challenging task. Here we used the mating differentiation in yeast *Saccharomyces cerevisiae* as a model and developed integrated experimental and computational approaches to analyze the proteomic dynamics during the process of cell fate determination. When exposed to a high dose of mating pheromone, the yeast cell undergoes growth arrest and forms a shmoo-like morphology; however, at intermediate doses, chemotropic elongated growth is initialized. To understand the gene regulatory networks that control this differentiation switch, we employed a high-throughput microfluidic imaging system that allows real-time and simultaneous measurements of cell growth and protein expression. Using kinetic modeling of protein dynamics, we classified the stimulus-dependent changes in protein abundance into two sources: global changes due to physiological alterations and gene-specific changes. A quantitative framework was proposed to decouple gene-specific regulatory modes from the growth-dependent global modulation of protein abundance. Based on the temporal patterns of gene-specific regulation, we established the network architectures underlying distinct cell fates using a reverse engineering method and uncovered the dose-dependent rewiring of gene regulatory network during mating differentiation. Furthermore, our results suggested a potential crosstalk between the pheromone response pathway and the target of rapamycin (TOR)-regulated ribosomal biogenesis pathway, which might underlie a cell differentiation switch in yeast mating response. In summary, our modeling approach addresses the distinct impacts of the global and gene-specific regulation on the control of protein dynamics and provides new insights into the mechanisms of cell fate determination. We anticipate that our integrated experimental and modeling strategies could be widely applicable to other biological systems.

## Author summary

A systematic characterization of the proteomic changes during the process of cell differentiation is critical for understanding the underlying molecular mechanisms. However, protein expression can be largely affected by changes in cell physiological state, which hampers the detection of regulatory interactions. Here we proposed an integrated experimental and computational framework to reconstruct regulatory circuits in mating differentiation of budding yeast *Saccharomyces cerevisiae*, in which distinct cell fates are triggered by alteration in pheromone concentration. A modeling approach was developed to decouple gene-specific regulation from growth-dependent global regulation of protein expression, allowing us to reverse engineering the gene regulatory circuits underlying distinct cell fates. Our work highlights the importance of model-based analysis of proteomic data and delivers new insight into dose-dependent differentiation behavior of budding yeast.

## Introduction

Retrieving gene regulatory networks from experimental measurements lies at the foundation for deciphering the mechanistic basis of cellular responses. To date, several strategies have been proposed to reconstruct biological networks. It is possible to infer network connectivity directly from genome-wide localization analysis, which takes advantages of high-throughput techniques to identify genomic sites bound by transcription factors (TFs) [1–4]. However, uncovering the physical interactions is insufficient to bring insight into the underlying regulatory mechanisms and recapitulate the dynamics of the system. Another strategy makes use of the simultaneous measurements of network elements and requires reverse engineering methods to deduce the network structure. A plethora of algorithms have been proposed to reconstruct gene regulatory networks in different organisms, and their performance has been quantitatively assessed [5–8]. Well-established methods include statistical methods based on correlation and mutual information [9, 10], ordinary differential equation (ODE) model [11], Bayesian networks [12] and Boolean network models [13, 14]. Prior knowledge about the organization of the biological network can be further incorporated into the workflow to facilitate the reverse engineering process [15, 16].

Despite these research achievements, several challenges exist in the reconstruction of biological networks. Gene expression profiles are widely used to retrieve transcriptional regulatory networks [8] with the implicit assumption that the activity of a TF is proportional to its mRNA level. However, the expression level of TFs is also subject to post-transcriptional regulations. Earlier studies employing simultaneous measurements of the transcriptome and proteome showed substantial differences between the mRNA and protein abundance either at the population level or the single-cell level [17–19]. On the other hand, although proteomic data provides a more reliable estimation of gene activity, it is not a good indicator of gene regulatory events. Physiological changes that involve global variations in ribosome number, metabolite concentration and growth rate can also affect protein synthesis and dilution, contributing to a layer of regulation that is independent of gene-gene interactions [18, 20–22]. This is especially important for investigating the gene regulatory networks underlying cell fate switches, in which distinct cell fates are often associated with very different physiological parameters (such as growth rate and biogenesis). Recently, several analyses applied dynamic modeling of protein life cycles to characterize the effect of different factors on the variations in protein abundance [23, 24], and their results indicated the critical role of kinetic modeling in decoupling the influence from different levels of regulation.

**Fig 1:**
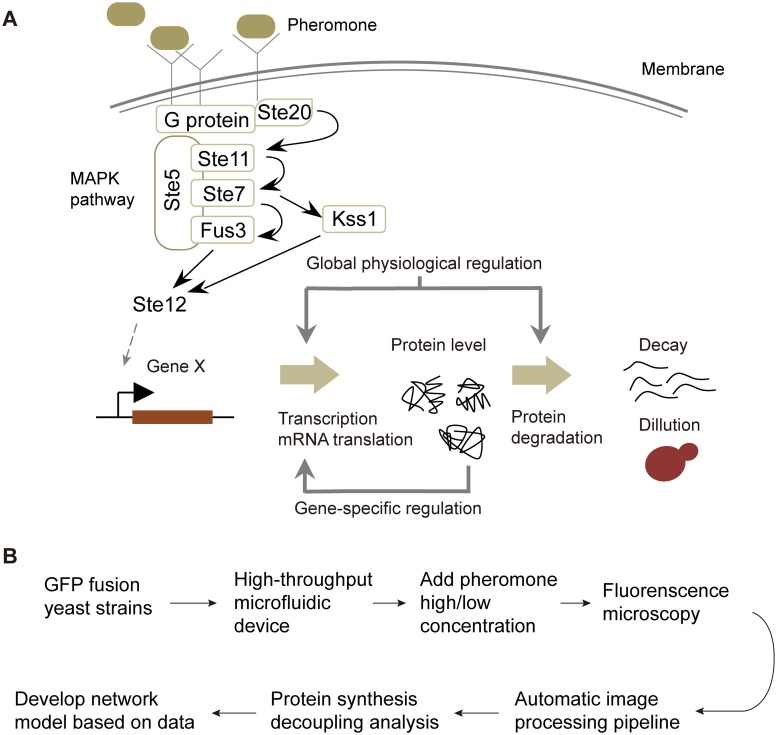
Protein expression control during the mating response and the analysis workflow. (A) Signal transduction and regulation of protein expression in the yeast mating response. The control of protein concentration is subject to two levels of regulation: genome-wide regulation relevant to the physiological response and gene-specific regulation, which was identified by our work. (B) Analysis workflow to uncover the regulatory circuits underlying distinct cell fates.

In this work, we incorporated high-resolution gene expression and cell growth profiling into kinetic modeling to study the cell fate determination in yeast mating response. The yeast mating response pathway is among the best-characterized models in the study of signal transduction, in which the external signal is transmitted through a mitogen-activated protein kinase (MAPK) cascade. This signal eventually activates transcription factor Ste12, which initiates downstream gene regulatory programs (Fig 1A). Despite a wealth of detailed information revealed by past studies [25], a less-studied perspective of the mating response is the cell fate decision governed by changes in the pheromone concentration [26–28]. While growth arrest and shmoo-like morphology is triggered when cells sense a high concentration of pheromone, yeast cells initiate a chemotrophic elongated growth along the pheromone gradient in response to a lower dose of pheromone. Due to the complexity of gene expression programs induced by pheromone stimulation, the mechanism underlying the mating differentiation switch remains elusive. Therefore, it represents a unique opportunity for quantitative investigations into whether and how divergent gene expression networks leading to distinct cell fates can be stimulated in a dose-dependent way.

Through kinetic modeling of protein abundance, we found that the observed protein dynamics in the mating response were partially determined by changes in the physiological state of the cell. Therefore, a model-driven framework named protein synthesis decoupling analysis (PSDA) was developed to decouple gene-specific regulation information from the influence of global physiological regulation. Based on the temporal order of gene-specific changes from PSDA, the putative regulatory networks were then reconstructed using a Boolean network model [14, 29]. These model analysis revealed network rewiring during cell differentiation and suggested a pheromone-dependent regulation of the TOR signaling pathway [30]. In summary, our results highlight the importance of considering the global physiological effects on gene expression control and provide mechanistic insights into the cell fate decisions triggered by different doses of pheromone.

## Results

### High-resolution temporal measurements of protein abundance and synthesis rate

To quantify the effect of physiological constraints on protein dynamic changes and reconstruct the gene regulatory networks in the yeast mating response, we developed a high-throughput microfluidic device that features a throughput of 96 experiments in one single run, continuous control of the medium and an automatic image processing pipeline. The system allows for simultaneous measurements of cell mass accumulation and protein expression level (Fig 1B). We used our platform to track the expression of ~200 fluorescently tagged genes as well as the growth dynamics of yeast cells in response to high and intermediate levels of pheromone (0.59 μM and 5.9 μM). These data offered a comprehensive view of the downstream gene regulatory response with unprecedented temporal resolution. In our experiment, the yeast strains from a green fluorescent protein (GFP)-tagged library [31] with *BAR1*+ background were cultured and loaded into a 96-well microfluidic device (S1A Fig), in which each strain was confined within the observation region of an insulated chamber for time-resolved analysis. The chemical condition of the chamber was controlled in an accurate and continuous way, and the concentration of pheromone was set to a high or intermediate level about 1 h after cell loading. In each experiment, phase contrast and fluorescence images of cells under pheromone stimulation were acquired at an interval of 5-min for 6 hours, producing >20000 images with single-cell resolution from a single chip. An image processing pipeline was adopted to automatically track the fluorescence intensity of each cell and estimate the growth rate via quantification of the accumulation of cell mass. To study the underlying mechanism of mating differentiation switching, we focused on a set of 195 selected genes including 79 Ste12 regulated genes [3], 52 transcriptional factors that are critical in the regulation of protein expression, and 64 manually selected genes that are representative of different functional groups relating to mating response.

**Fig 2:**
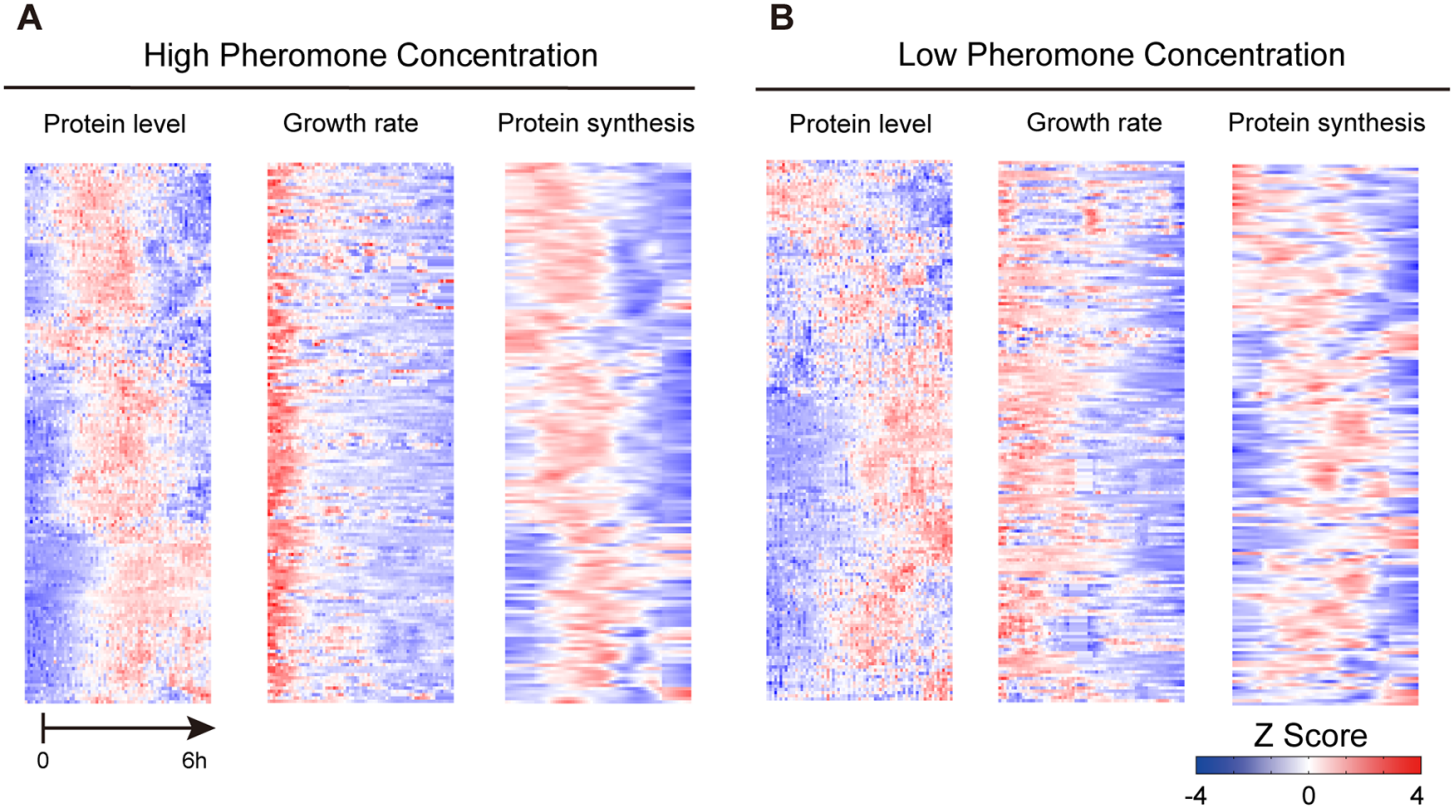
High-resolution profiling of protein expression and synthesis rates. (A) Protein expression, growth rate and protein synthesis rates for the investigated genes in yeast cells in response to a high concentration of pheromone. The order of the genes in the heatmap is determined via hierarchical clustering of the gene expression profiles. Values are normalized and transformed to a z-score. (B) The normalized values of protein expression, growth rate and protein synthesis rate for genes in yeast cells exposed to the intermediate concentration of pheromone.

We monitored the protein expression and growth dynamics of the selected yeast strains from the GFP library in response to two different concentrations of pheromone (first two columns of Fig 2A and 2B, S1 Dataset). Consistent with previous studies [26, 27], different cell fate responses, characterized by distinct morphological changes were induced depending on pheromone levels (S1B Fig). When exposed to a high concentration of pheromone, cells underwent a sudden cell cycle arrest within 60 min and formed multiple short projections. Intriguingly, the protein abundances of most examined genes were first up-regulated and then relaxed to their original levels towards the end of the experiment (Fig 2A, left column). These results exhibit a substantial deviation from the transcriptional regulation reported before, because previous microarray data showed that a reduction in the transcript levels of at least 48% of examined genes under the same condition [32]. In addition, we observed the growth rate of shmooing cells underwent a sudden arrest, falling to about half of its normal value within 60 min (Fig 2A, middle column). Under an intermediate level of pheromone stimulation, yeast cells arrested in cell cycle synchronously and initiated a chemotrophic elongated growth. Accordingly we observed a gradual slowdown of growth rate, in accord with the linear mode of cell mass accumulation in elongated cells (Fig 2B, middle column). The protein abundances of most examined genes were also up-regulated, but to a lesser extent and were more fluctuating over time (Fig 2B, left column). These results suggest that cell cycle progression and cell growth may influence the protein dynamics, in accordance with previous studies [33].

To calculate the actual protein synthesis rate from our data, we next employed a mass-action kinetic model of protein abundance and investigated the interdependence between the measured fluorescence and the growth dynamics. In this model, the changes in protein concentration were considered to be due to protein synthesis and decay; the latter was attributed to protein degradation and cell growth dilution (see Methods). To calculate the synthesis rate for each protein, we took into account the expression *P(t)* and growth profiles *γ*(*t*) generated from the time-resolved measurements (first two columns of Fig 2A and 2B) and the protein degradation rate *d* obtained from a genome-wide analysis of protein half-lives [34]. The kinetic model is solved in a discrete manner so that the protein concentration change between two time points can be expressed as Δ*P*(*t*) = Δ*tα*(*t*) − Δ*t*(*d* + *γ*(*t*))*P*(*t*). The calculated results of protein synthesis rate *α*(*t*) are presented in the third columns of Fig 2A and 2B. Under the high pheromone level, the synthesis rates of most examined proteins were slightly up-regulated followed by a significant decrease to a much lower level. Because the average mRNA level of examined genes is not significantly reduced throughout the same time period [32], our results suggest that the genome-wide regulation of translation rate is responsible for the global decrease in protein synthesis rate. This global reduction in translation rate may compensate the decrease in protein dilution rate caused by cell cycle arrest and lead to an overall decline of protein abundance after the initial induction (Fig 2A, right column). In contrast, in cells responding to an intermediate level of pheromone, the protein synthesis rate was more fluctuating with a general trend of a more prolonged increase (Fig 2B, right column). These results showed that distinct cell fates are associated with different dynamic changes in protein synthesis rate, as protein biogenesis might be strongly inhibited in the shmooing cells but not in the elongated growing cells.

### Estimation of gene-specific regulation through dynamic modeling

**Fig 3:**
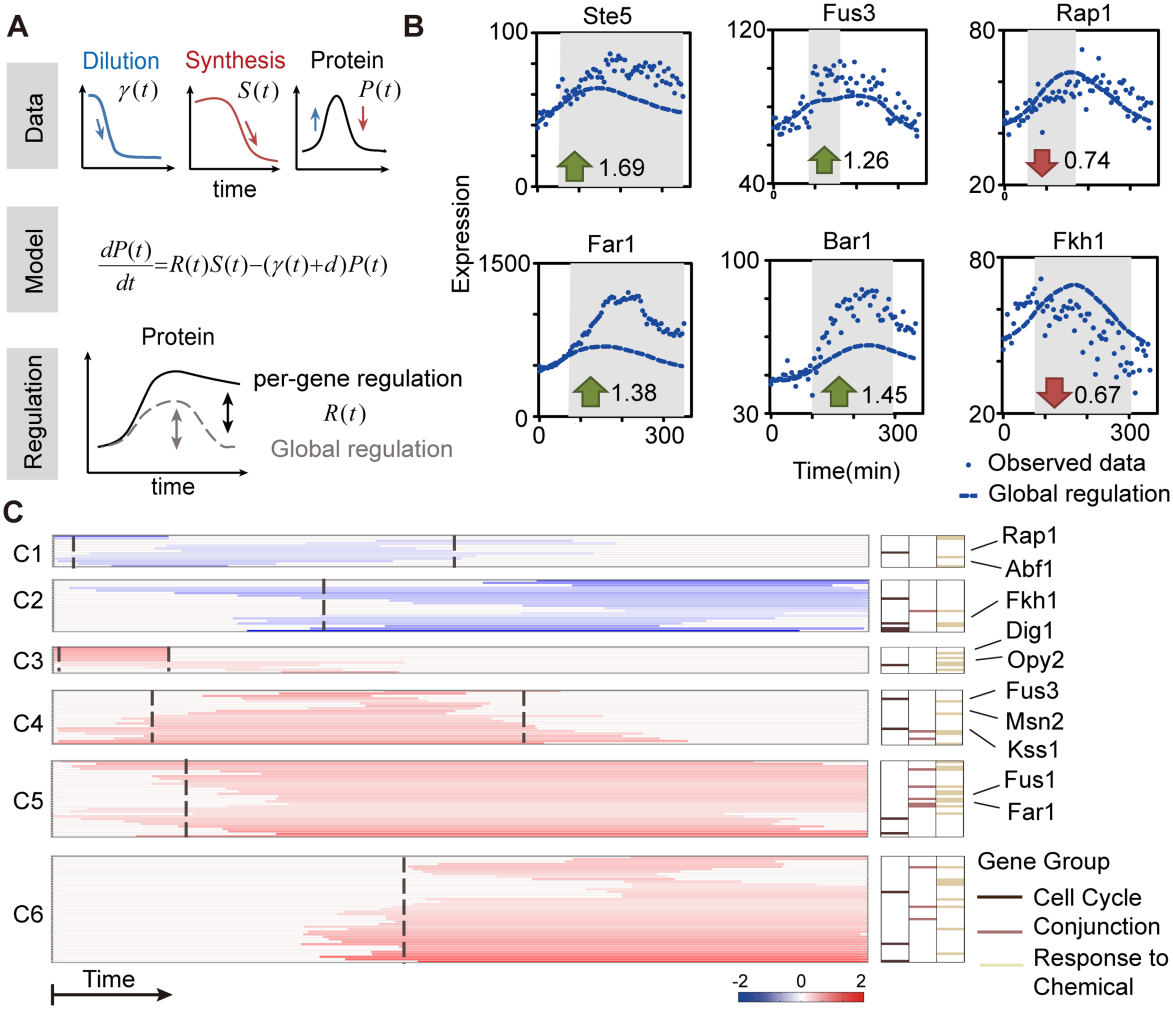
Mechanism of PSDA and identified regulation events in shmooing cells. (A) PSDA deconvolutes the gene-specific regulation by modeling the effect of the physiological response of the yeast cells. (B) Examples for PSDA results of six different strains. The dashed line indicates the protein dynamic changes caused by global regulation and the dots represent the observed protein abundance. Time window of gene-specific regulation are gray shaded. Fold change of regulation is indicated; green arrow (activation), red arrow (inhibition). (C) Identified gene regulation events in shmooing cells. Values are the log-transformed fold change of the regulation level. Distinct temporal modes were distinguished by k-means clustering, and the 50% activation/inhibition time for each cluster is represented by the dashed line. C1-C6 denote clusters 1–6. Right column, proteins responsible for the cell cycle (dark brown), conjunction (brown) and chemical response (light brown).

Since gene-gene interactions and variations in physiological parameters can affect protein dynamics at the same time, we investigated their impacts on protein expression control individually through kinetic modeling. We first examined to what extent the global physiological regulation inherent to each cell strain accounted for the dynamic changes in protein expression. We used a similar kinetic model as that used in the estimation of protein synthesis rate. The only difference is that the synthesis rate of each protein was replaced by a rescaled control rate *S(t)* generated as follows: (1) we estimated the dynamic changes of global protein synthesis rate for two phenotypes, which revealed the physiological constraints on protein expression control in the pheromone response (S3 Fig); (2) the global synthesis rate of each protein was generated by rescaling the normalized control rate to model the differences in their basal expressions. The rescaling factor is proportional to the steady state protein abundance prior to pheromone stimulation. The protein dilution and degradation rates were set to the per-strain values. Therefore, by estimating changes in protein synthesis and dilution that are independent of gene-specific interactions, we were able to obtain the protein dynamic changes caused by global regulation. We found the simulated protein abundance profiles showed a trend of transient up-regulation even though there is no gene-gene interactions (Fig 3A, S3 Fig). The Pearson correlation between the predicted and observed fold change was 0.37 (p-value = 8.5 x 10^−6^), indicating that about 14% of the variance in the protein level can be explained by the influence of physiological factors. Thus our results suggest that although physiological regulation is responsible for the global trend of protein dynamics, the protein dynamics of different genes are mainly determined by gene-specific regulations.

We assume that the discrepancy between the measured expression and the above-mentioned calculation stems from gene-specific regulation. To quantitatively dissect the gene-specific transcriptional regulation, we developed a computational framework named Protein Synthesis Decoupling Analysis (PSDA) that employs a robust and cross-grain estimation of the time window and fold change of gene regulation events (Fig 3A). We used a modified kinetic model of protein life cycle, in which protein synthesis is the product of the gene-specific regulatory term *R(t)* and the global synthesis rate *S(t)* related to the physiological status. Therefore, rate parameters except *S(t)* contributed to the global regulation of protein abundance and *R(t)* was used to quantitatively assess the protein dynamic changes that could not be explained by the global regulation for each gene, thereby identifying gene-specific regulation dynamics. To filter out the noise inherent to experimental data and capture the main features of gene regulation, the gene-specific regulation term *R(t)* was parameterized as a pulse-like function [18, 24]. The parameters were estimated utilizing a global optimization algorithm, namely differential simulated annealing (DSA) [35]. In each round of parameter evaluation, we simulated the dynamics of protein concentrations and set the value of the objective function to the sum of squared errors between the simulated and observed trajectory. The Markov chain length was set to 100 and the maximum round of function evaluation was set to 2000 for each gene. Distinct modes of regulation were deconvoluted from similar dynamic patterns of protein abundance in different cell fate responses (S2 Dataset). Notably, our algorithm is capable of identifying the transcriptional down-regulation of genes despite of the induction of overall protein expression that is purely due to the alterations of physiological parameters. For instance, although the expression of *FKH1* showed a 1.5-fold induction in cells undergoing shmoo formation, PSDA revealed a reduction in its protein synthesis in the last 4 h. This reduction in the protein abundance of Fkh1, which is a cell cycle regulator, could account for the cell cycle arrest observed under this condition (Fig 3B).

Our PSDA approach yielded the gene-specific dynamics that are consistent with previous microarray results [32] (S4 Fig), for most genes examined in this study. For example, synthesis of *BAR1* is up-regulated after 1.5 h (Fig 3B, gray shaded window in the panel labeled “Bar1”), contributing to a delayed negative feedback that modulates the response duration. Our analysis also revealed up-regulation of genes in the MAPK cascade, such as *FUS3* and *STE5*, consistent with previous microarray data [32] (Fig 3B, gray shaded window in the panels labeled “Fus3” and “Ste5”). More importantly, the PSDA approach can identify time intervals of regulatory events that are otherwise invisible from the protein expression profiles. For instance, we found synthesis of *FUS3* was up-regulated during the intermediate phase, while several typical mating genes, such as *FAR1*, were induced in the late phase of experiment (Fig 3B, gray shaded window in the panels labeled “Fus3” and “Far1”). Therefore, PSDA can identify gene-specific regulatory events from protein expression profiles and reveal the temporal patterns of regulatory events in a quantitative manner.

### Reconstruction of the gene regulatory network for shmoo formation

To further characterize the regulatory modes of cells committed to the shmooing fate, we clustered genes into 6 groups according to their temporal patterns of gene-specific regulation, with 2 groups inhibited and 4 groups activated (Fig 3C; S5A Fig). Using a smaller cluster number makes it hard to detect the sequential gene regulatory steps. For example, reducing the cluster number could merge C3 and C4 into a new cluster and we cannot distinguish genes that are activated in the early phase form that activated in the intermediate phase. On the other hand, subdividing the genes into more clusters would increase computation complexity and the number of possible networks, but does not alter the general network topology (see Discussion below). The first cluster includes RP genes such as *RPL1B* and TFs, such as *ABF1* and *RAP1*, which are important regulators of RP synthesis [36]. The synthesis rate of these genes is rapidly down-regulated before recovering to the pre-treatment value, which might lead to decreases in ribosome synthesis (Fig 3C, C1). The second cluster contains genes that are inhibited with a time lag of ~2 h. This cluster is enriched with cell cycle genes (S1 Table), including *FKH1/2*, which are TFs that mediate the expression of genes in M phase (Fig 3C, C2). Genes in cluster 3 show a rapid and transient induction of expression and are primarily involved in stress-activated signaling responses, e.g., *OPY2* in the high-osmolarity glycerol (HOG) pathway and *MDS3* that associates with the TOR pathway. *DIG1*, an important regulator of the pheromone response pathway, is also included in this cluster (Fig 3C, C3). Genes encoding the components of MAPK pathway, such as *KSS1* and *FUS3* are enriched in cluster 4 and are transiently induced after cluster 3 genes (Fig 3C, C4). Cluster 5 includes the genes participate in execution of yeast mating and fusion, such as *FUS1* and *FAR1*. These genes are up-regulated slightly later than cluster 4 genes and exhibit a more prolonged induction patterns (Fig 3C, C5). Finally, cluster 6 genes, such as *ISW1* and *SNF2*, are up-regulated very late in the response and are primarily involved in chromatin remodeling (Fig 3C, C6).

**Fig 4:**
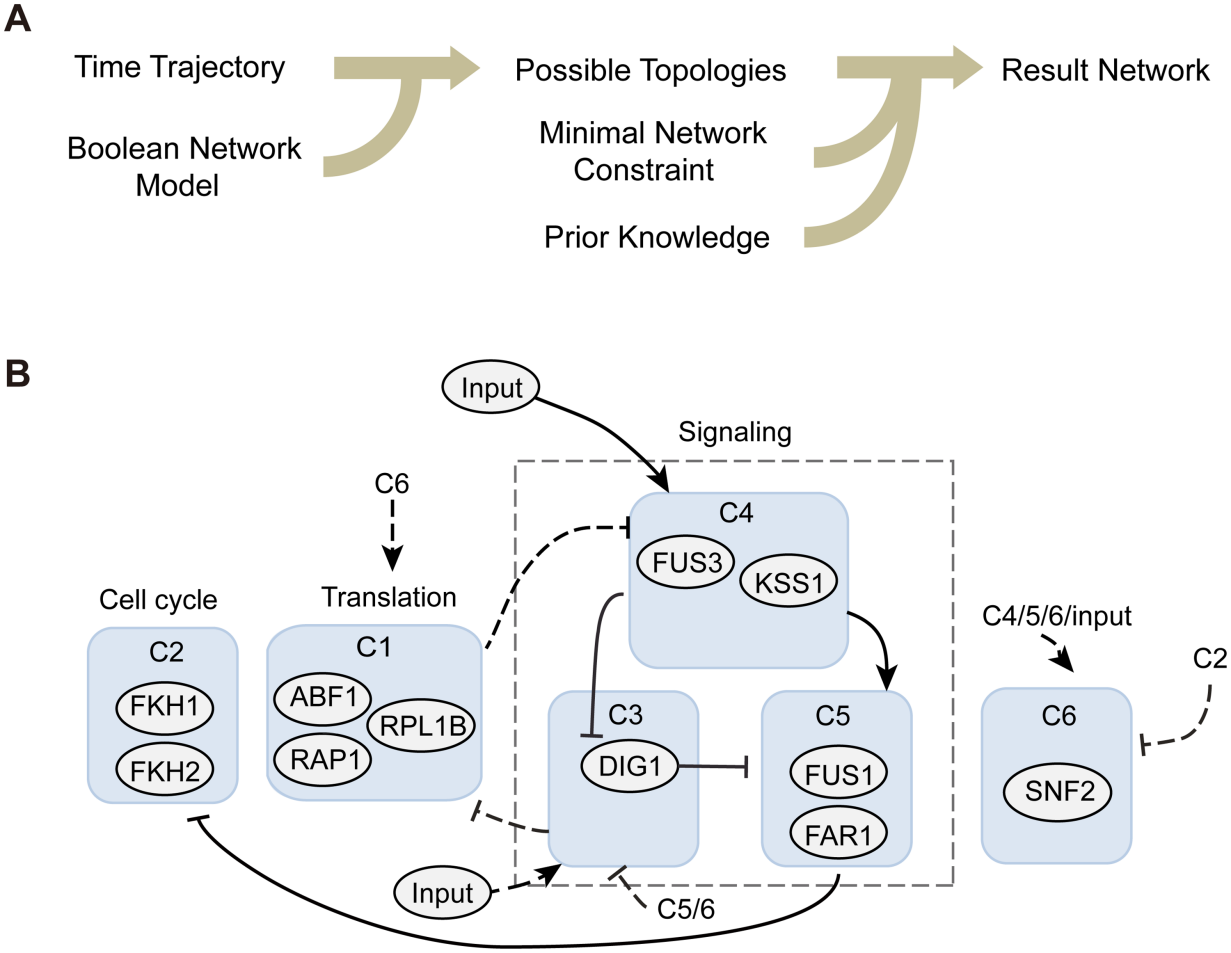
Reconstruction of the regulatory circuit responsible for shmoo formation. (A) Workflow of the reverse engineering process. A Boolean network model was used to deduce the constraints on the network interactions from the discrete time trajectory. After generating all possible networks, the minimal network constraint and prior knowledge about signal transduction were incorporated to select for minimal circuits responsible for the shmoo formation. (B) The resulting regulatory circuit. Solid and dashed edges are used to denote the canonical pheromone response pathway and novel regulations, respectively. Edges with bar-end, regulation of inhibition; edges with arrow-end, activation. The input of the network is the TFs activated by pheromone that can directly regulate gene expression, such as Ste12 and Tec1.

Because genes in the same cluster show similar temporal patterns, we hypothesized that they might share same upstream regulators. So we treated each cluster as a ‘meta-gene’ and generated its activation/inhibition time sequence via a threshold model (S5B Fig), which allows us to reconstruct the putative interactions among the clusters by analyzing the time trajectory using a Boolean network model [14, 16]. A wide range of parameter values was used in the threshold model to investigate the robustness of our results (S5B Fig). The discrete trajectory resulted in 4.1 x 10^8^ possible networks, including 96 minimal networks without redundant edges. We further incorporated prior knowledge to further confine the network structure (Fig 4A). The regulatory network and representative genes in each cluster are illustrated in Fig 4B. The “input” of the network is the TFs activated by pheromone that can directly regulate gene expression, such as Ste12 and Tec1. The reconstructed network successfully captures the core structure of pheromone response pathway and reveals genetic interactions in accordance with previous knowledge of the system (Fig 4B, solid arrows). The network structure is robust to the choice of the cluster number. Subdivision of the largest cluster (C6) into smaller clusters would increase the number of possible networks, while leaving the general network structure unchanged (S6 Fig). More importantly, our results also suggested novel putative interactions in the gene expression programs induced by pheromone (Fig 4B, dashed arrows). For example, the stress response genes in cluster 3 might repress translation by inhibiting cluster 1 genes. Additionally, in the early phase of the response, cluster 2 cell cycle genes may repress the expression of cluster 6 genes, implying a coordinated regulation of cell cycle and chromatin remodeling during the mating response. In summary, these results support the use of our experimental and computational approaches in dynamic network analysis and imply the potential interactions of the canonical mating pathway with various signaling and cellular processes, offering a more comprehensive picture of the yeast mating response.

### Dose-dependent network rewiring underlies mating differentiation

**Fig 5:**
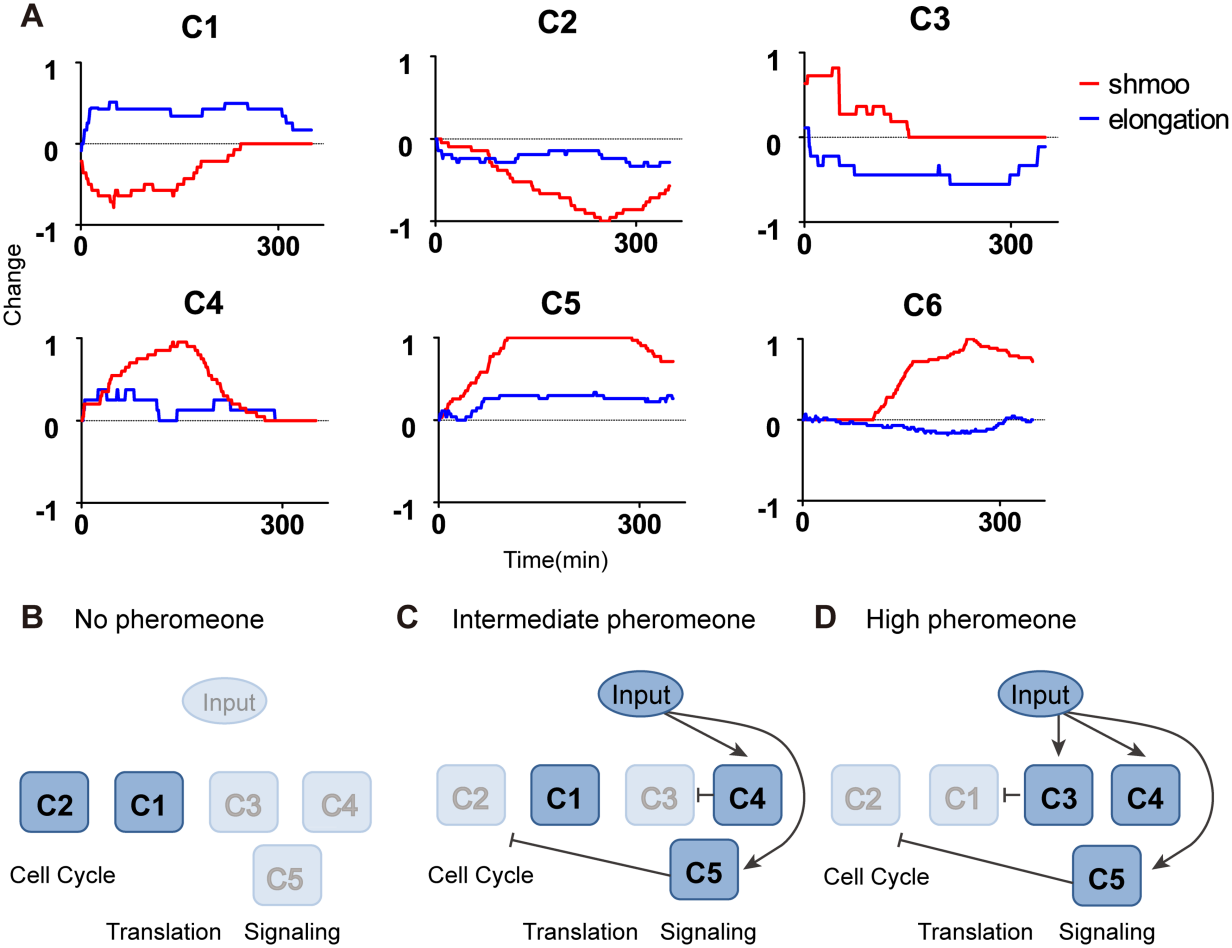
Dose-dependent regulation patterns and mechanism for cell fate determination. (A) Regulation modes of the 6 gene groups in elongated cells (colored in blue), and in shmooing cells (colored in red). The activity of each cluster was averaged from all of the gene-specific regulation information for the corresponding genes, with regulation level normalized to 1/-1 for activation/inhibition. Several clusters show divergent behaviors in the two phenotypes, as in observed for C1 and C3, which contributes to the differential regulation that determines the cell fate switching. Schematic illustration of active gene groups and regulatory circuits responsible for different cell fates, including vegetative growth (B), elongated growth (C) and shmooing (D). Edges that result in the activation/ inhibition of gene groups are illustrated.

To investigate the mechanisms underlying dose-dependent mating differentiation, we next reconstructed the gene regulatory network for cells committed to chemotropic elongated growth in response to the intermediate pheromone level. To this end, we examined the deconvoluted gene-specific regulations of the six meta-genes (classified in Fig 3C) for elongated growth and compared their time trajectories with those of the cells committed to shmoo formation upon a high pheromone dose. Interestingly, although we observed striking differences in protein dynamics of all six meta-genes between the two cell fates, only the genes in cluster 1 and 3 exhibited a qualitative difference. Cluster 1 genes are down-regulated during shmoo formation in response to the high level of pheromone stimulation, but are up-regulated during elongated growth in response to the intermediate pheromone dose. In contrast, cluster 3 genes are transiently induced during shmoo formation, but are repressed during elongated growth. All the other clusters showed quantitative differences between the responses to different doses of pheromone, in which the gene regulation are towards the same direction and only differ in the extent of changes (Fig 5A).

Based on the dynamic changes of the meta-genes in response to the intermediate level of pheromone, we generated a putative regulatory network that can recapitulate the protein dynamics during elongated growth (S7A and S7B Fig). We then compared this network structure with that of shmoo formation response (Fig 5C–5D). Consistent with the analysis of protein dynamics in Fig 5A, we found that the core network structure consisting of the interactions between clusters 2, 4 and 5 remained unchanged in the two cell fates. The major difference lies in cluster 1 and 3. During elongated growth in response to an intermediate pheromone level, the RP genes in cluster 1 are induced but the stress response genes in cluster 3 are repressed. In contrast, during shmoo formation triggered by a high pheromone dose, cluster 1 genes are inhibited whereas cluster 3 genes are induced. Furthermore, since cluster 3 undergoes a slightly faster response than cluster 1 during shmoo formation, it is possible that the stress response genes in cluster 3 may contribute to the repression of RP genes in cluster 1. To examine the robustness of the uncovered networks, we further investigated the attractor landscape for cell fate determination in the mating response (S7C and S7D Fig). Although re-wiring of the regulatory network alters the attractor landscape, the two biological phenotypes emerge as the largest attractors, revealing the dynamic robustness of the cell fate commitment. Taken together, our modeling analysis suggested that the dose-dependent regulation of stress response genes might lead to the phenotypic differences associated with distinct cell fates, in which the cells undergoing elongated growth have an enhanced cell growth and biogenesis capacity, whereas the cells committed to shmoo formation feature a reduced biogenesis capacity.

### Identification of a putative pathway crosstalk during mating differentiation

**Fig 6:**
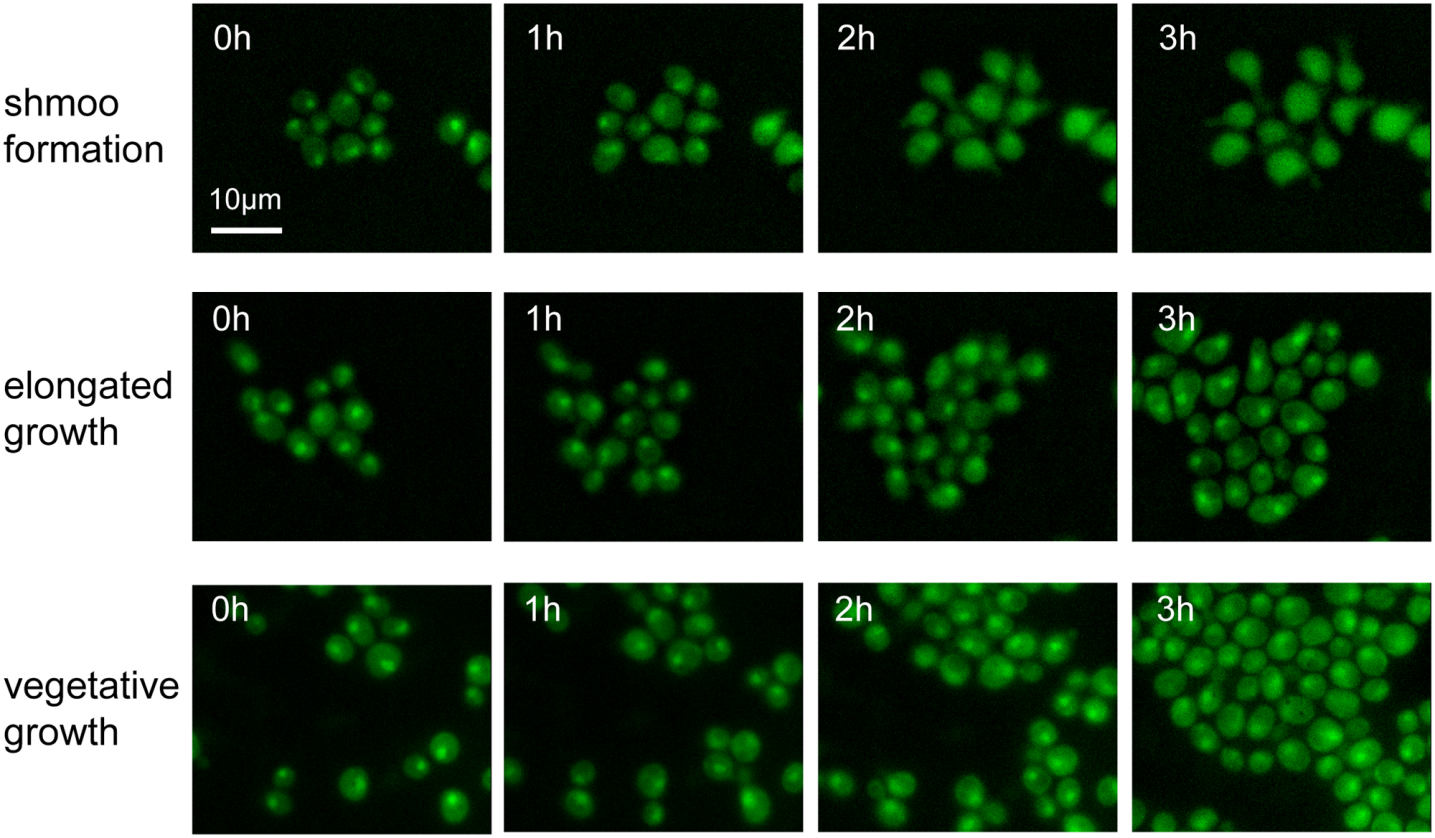
Pheromone-dependent translocation of Sfp1 suggests a dose-dependent crosstalk between the pheromone response pathway and the TOR-regulated ribosomal biogenesis pathway. Time course of Sfp1-GFP localization in cells committed to different cell fates, as indicated.

To further validate the model results, we experimentally explored the molecular connections between the pheromone response pathway and the RP or stress response genes. In *S*. *cerevisiae*, induction of stress response genes and repression of RP genes are simultaneously associated with the responses to nutrient limitation or stresses and are mediated by the general stress responsive pathways, such as TOR or protein kinase A (PKA) pathways [37, 38]. Thus, we examined major stress responsive regulators in yeast, including Sfp1—a stress responsive transcription factor [30], Hog1 –a stress-activated protein kinase [39], Msn2 –a general stress responsive TF in the PKA pathway [40], Yap1 –a TF involved in transcriptional response to various stresses [41], and Tpk1 –the catalytic subunit of PKA [42]. These regulators can govern the expression of RP and/or stress response genes and their activities are primarily controlled via nucleocytoplasmic translocation. We found that pheromone stimulation had no effect on the localization of Hog1, Msn2, Yap1 or Tpk1 (S7 Fig). In contrast, whereas localized in the nucleus under vegetative or elongated growth conditions, Sfp1 translocated from the nucleus to the cytoplasm in response to a high level of pheromone stimulation leading to shmoo formation (Fig 6; S8 Fig; S1 Video). Intriguingly, Sfp1 has been best known as a stress responsive TF primarily involved in the TOR signaling pathway [30, 43]. Under optimal growth conditions, the Tor kinases are active and Sfp1 localizes in the nucleus where it activates the expression of RP and ribosomal biogenesis genes; in response to nutrient limitation or chemical stress, Tor kinases are inhibited and Sfp1 is translocated from the nucleus to the cytoplasm. Therefore, our results suggest a potential cross-regulation of the TOR-dependent ribosomal biogenesis pathway that only occurs in shmooing cells. This pathway crosstalk could serve as a molecular switch that mediates the dose-dependent network rewiring and cell fate determination during mating differentiation.

## Discussion

It is a challenging task to resolve regulatory networks from experimental observations in eukaryotic organisms, despite continuous advances in reverse engineering methods in the past decades [8]. One reason for this is that regulation of protein abundance is not only determined by the gene-gene interactions, but also subject to the availability of cellular resources and changes in growth dynamics. In this paper, we employed an integrated approach that combines high-throughput experiments, dynamic modeling of the protein life cycle and a reverse engineering method to investigate the regulatory mechanisms underlying distinct cell fates in the yeast mating response. Based on the high-resolution profiling of the protein abundance and growth dynamics of yeast cells, we found that gene expression can be strongly affected by the global changes in cell physiological state, hampering the identification of gene-specific regulation events that are critical for network discovery. Here, we solved this problem by taking a model-driven approach (PSDA) to assess the influence of global regulation and deconvolute the gene-specific regulation, which enables us to further reconstruct regulatory networks underlying different mating responses.

Our PSDA method confers advantages of robust detection of regulation events and little data requirement, but is not without limitations. For example, we assumed the regulation of protein level mainly takes place in the synthesis part. This assumption, supported by previous studies [23, 44], helps to reduce the number of free parameters while preserving the capacity to identify regulatory events. However, it may overlook dramatic changes in protein degradation that is crucial for some specific responses. Also PSDA quantitates the summarized effect of regulations in transcriptional and translational levels and therefore it cannot distinguish between the two effects. Further incorporation of time-resolved transcriptome data may enable more accurate quantification of transcriptional and translational regulation and comparison with relevant methods [24].

The putative regulatory networks were generated without empirical parameterization of the regulation functions, yet successfully recapitulated the dynamics of the system underlying different phenotypic responses. Our reconstructed network includes genes encoding the components of canonical pheromone response pathway that regulate signal transduction, cell cycle arrest and cell fusion. In addition, our network analysis suggests a conditional regulation of RP and stress response genes, which guided our identification of the dose-dependent translocation of a stress responsive transcriptional factor, Sfp1. Since the localization of Sfp1 is directly regulated by the activity of Tor kinases [30, 45], it is possible that Tor activity is only diminished in shmooing cells, but not in elongated cells. Intriguingly, a previous study [33] showed using microarray time course analysis that a high dose of pheromone down-regulates 54 ribosomal proteins. This down-regulation is independent of pheromone-induced cell cycle arrest and the kinetics is similar to that observed in response to rapamycin treatment. Their findings are consistent with our modeling prediction and experimental results, pointing to a possible cross talk between the mating pathway and the TOR pathway. A systematic epistasis analysis of the pheromone response pathway would provide us further insights into the mechanisms of this pathway crosstalk.

Taken together, our experimental and computational approaches represent an integrated framework for analyzing the proteomic dynamics during cell differentiation. Given the growing interests in large-scale protein dynamics and network analysis, we anticipate that our strategies would be widely applied to enable systems-level understanding of other biological systems.

## Materials and methods

### Strain, media and cell preparation

The yeast cell strains used in this study were selected from a chromosomally GFP-tagged library. Strains were grown to saturation at 30°C and further diluted and cultured for another 8 hours to reach the exponential growth phase before the microfluidic experiments. The alpha factor (Sigma-Aldrich, St. Louis, MO) level was set to 5.9μM/L for the high concentration and 0.59μM/L for the intermediate concentration.

### Microfluidics device and microscopy

A high-throughput microfluidic chip was used in our fluorescence experiment, which allows a maximum of 96 parallel experiments across 2 different conditions (S1A Fig). Our chip was fabricated with PDMS (polydimethylsiloxane, RTV615, Momentive Performance Materials Inc.) using standard soft lithography technology. Each strain was loaded into an individual channel and a constant flow rate of 400 μl/h for fresh media was achieved. Phase contrast and fluorescence images of yeast cells were generated via a Nikon Ti-E microscope in combination with NIS-Elements software every 5 min. A cell culture incubator around the microscope was used to maintain a temperature of 30°C.

### Estimation of protein synthesis rate and PSDA

We developed a kinetic model of protein dynamic change in which:

$$\frac{dP(t)}{dt}=\alpha (t)-(d+\gamma (t))P(t)$$

where *P*(*t*) is the protein concentration at time t, *α*(*t*) represents the protein synthesis rate and *γ*(*t*) denotes protein dilution rate, which equals the exponential cell mass accumulation rate. *d* is the protein degradation rate estimated from a genome-wide measurement of protein half-lives [34].

We suspected that the per-gene regulations in the response mainly took place in the protein synthesis term. Our kinetic model for protein concentration was thus modified to the following form:

$$\frac{dP(t)}{dt}=R(t)S(t)-(d+\gamma (t))P(t)$$

in which *S*(*t*) is the global synthesis rate that reflects the changes of cellular resources related to protein synthesis, and *R*(*t*) is the temporal regulation term of the protein. We adopted an efficient parameter optimization algorithm named differential stimulated annealing (DSA) to estimate the regulation parameters for each protein [35]. The calculation was based on a customized MATLAB version of the algorithm. For detailed information, see S1 Text.

### Boolean network model

In the Boolean network model, each node represents a biological species. *S*_*i*_(*t*)∊{0, 1} is used to denote the state of node i at time t. Regulation from node j to node i is represented by the coefficient *a*_*ji*_, which is positive for activation and negative for inhibition. Update of the node states is described by the following Boolean functions:

$$\{\begin{array}{c} S_{i}(t+1)=\theta (\sum_{j}S_{j}(t)a_{ji}),\sum_{j}S_{j}(t)a_{ji}\neq 0\\ S_{i}(t+1)=S_{i}(t),\sum_{j}S_{j}(t)a_{ji}=0 \end{array}$$

where *θ* is the Heaviside step function as follows: *θ*(*x*) = 1 when x > 0 and *θ*(*x*) = 0 when x < 0. From a given initial state, the state of the system is updated until it reaches a steady state known as an attractor.

## Supporting information

S1 Text Methods. (DOCX)

S1 Table Percentage of gene ontology terms in the clusters. (DOCX)

S2 Table Time trajectory of the system under high concentration of pheromone. (DOCX)

S3 Table The rigid and interchangeable edges of all the nodes in shmoo formation. (DOCX)

S1 Video Time course of Sfp1-GFP localization in cells undergoing shmoo formation. (AVI)

S1 Dataset Protein expression and cell growth data for the investigated genes. (XLSX)

S2 Dataset PSDA results for the investigated genes. (XLSX)

S1 Fig Microfluidic device and image processing pipeline. (A) The image processing pipeline. The microfluidic chip allows maximumly 96 parallel experiments. The observation chamber in each channel is about 4 μm high and 200 μm wide. Growth medium was loaded into the chip from injection syringes driven by pumps and GFP-tagged strains were loaded through the wells by the side. The fluorescence microscope scanned over the chip every 5 min to generate phase contrast and fluorescence images of the yeast cells. The phase contrast images were used in cell segmentation, which provide basis for measuring GFP concentration and estimation of cell growth rate. (B) Microfluidic experiment reproduces different phenotypes of yeast cells in mating response. When exposed to high level of alpha factor, the yeast cells formed small projections sequentially to search for mating partner in the surrounding area. Under a lower dose of pheromone, the cells arrested their cell cycles and began chemotrophic growth, usually resulting in a radial pattern of colony.
(TIF)

S2 Fig Global protein synthesis rate for cells in response to different concentrations of pheromone. The global protein synthesis in shmooing response was averaged over the protein synthesis rate for genes that were not significantly regulated as observed in a former study [28], including YGL252C, YNL027W, YDL080C, YKL222C, YBR289W and YML099C. The resulting profile is also in agreement with the median trajectory of all the protein synthesis rates. The protein synthesis shows a ~40% reduction after slightly up-regulation in the first two hours, which may result from the combined effect of global inflation of protein abundance and reduction of RP mRNAs. In elongated cells, the global protein synthesis rate was estimated by averaging the protein synthesis rate for all the proteins. There’s no significant reduction in protein synthesis, indicating a norm rate of cell mass accumulation. Standard derivatives are illustrated in the Fig, with N = 162 for shmoo and N = 171 for elongation.
(TIF)

S3 Fig Dynamic of grow rate and protein expression of two yeast strains. The measured growth rate of two different yeast strains in shmoo formation, in which GFP is tagged to RIC1 (A) and STE2 (B). GFP concentration for the strains (dot) and protein dynamic profiles predicted from global regulation (dashed line) are illustrated in (C, D) respectively. The discrepancy of measured and predicted profile reveals the time window and relative level of per-gene regulation, with STE2 activated in the latter phase and no significant regulation for RIC1.
(TIF)

S4 Fig Expression of up- and down-regulated genes identified by PSDA. **(A)** Expression of up- and down-regulated genes in shmooing cells. Average expression value was generated by reprocessing data from [28], in which bar1 mutant cells were used. Data point with largest divergence between up- and down-regulated genes was underlined. (B) Expression of up- and down-regulated genes in elongated cells.
(TIF)

S5 Fig Robustness of the clustering results and discretization. (A) Hierarchical clustering and k-means clustering of gene-specific regulations in shmooing. Temporal modes of gene regulation events of 141 genes in shmooing cells was clustered by two methods. By introducing a threshold in hierarchical clustering and aligning the corresponding clusters of two methods, we found that the clusters identified by k-means clustering either recapture, or rearrange the clusters from hierarchical clustering, thus providing a more balanced dividing of the genes. (B) Robustness of the threshold model in discretizing time trajectory. The discrete time trajectories were generated by using different thresholds, i.e., 50%, 30% and 70%. Different colors denote different clusters. The relative sequence of regulation events were conserved in respect to the variations in threshold.
(TIF)

S6 Fig Result of introducing more clusters. (A) Hierarchical clustering of C6 results in 3 sub-clusters with different expression program in latter phase of mating response. (B) Discrete time trajectory for the 8-node network. New states relative to S2 Table is shaded in blue. (C) The resulting 8-node network. Addition of 2 clusters leads to new edges (green) but doesn’t alter the topology of the original network.
(TIF)

S7 Fig The regulatory circuit for elongation growth and attractor landscapes for two cell fates. (A) Gene regulatory circuit for elongated cells. Solid and dashed edges are used to denote the canonical pheromone response pathway and novel regulations, respectively. Edges with bar-end, regulation of inhibition; edges with arrow-end, activation. (B) Time trajectory of the regulatory circuit. The trajectory was generated by using the regulatory network in elongated cells, in which 3 edges were altered in respect to the differential regulations of six clusters. The initial state was changed due to the different regulation modes of C1 and C3 in elongated cells. The time trajectory recapitulates the dynamic changes of different clusters in elongated growth, as shown in Fig 5A. (C) Attractor landscape for shmoo formation. For 6-node Boolean network, there’re 2^6^ = 64 possible states (gray point). We selected a minimal network from Fig 4B to calculate *S*_*i*_(*t*+1) from a give state *S*_*i*_(*t*). Dynamic flow from one state to another is indicated by the directed arrow, with green arrows representing the biological trajectory in shmooing cells (S2 Table). Most of the states (gray points) converge to the biological state (1, 0, 0, 0, 1, 1, 1) (green point) of shmoo. Input value was set to 1 at all times. (D) Attractor landscape for elongated growth. Network from S7A Fig was used to update the state of network. We assume there’s a self-inhibition edge for cluster 6 to keep it in the inactivated state. Green point indicates the attractor in elongated growth, i. e. (1, 0, 0, 1, 1, 0, 1).
(TIF)

S8 Fig Localization of stress response genes in elongated and shmooing cells. Images of the same live cells were captured after 4 hours of pheromone addition by fluorescence microscopy. The subtract background filter of ImageJ (US National Institutes of Health) was applied with a rolling ball radius of 30 pixels to remove background noise.
(TIF)
