# Supplementary material for: Reconstructing the regulatory circuit of cell fate determination in yeast mating response
Source: PLoS Comput Biol. 2017 Jul 24;13(7):e1005671. doi: 10.1371/journal.pcbi.1005671 (PMC5546706; doi:10.1371/journal.pcbi.1005671)
Supplement: S1 Text — (DOCX) [file pcbi.1005671.s001.docx]

**S1 Text Methods**

**Strain, Media and Cell Preparation**

The yeast cell strains used in this study were selected from a chromosomally GFP-tagged library, which was constructed by incorporating the GFP tag into the C-terminal region of the genes through homologous recombination [1]. The successfully transformed cells contain a plasmid expressing His5+, and SD medium (his-) was used for culturing the yeast cells. Strains were grown to saturation at 30 ℃ and further diluted and cultured for another 8 hours to reach the exponential growth phase before the microfluidic experiments. The alpha factor (Sigma-Aldrich, St. Louis, MO) level was set to 5.9 μM for the high concentration and 0.59 μM for the intermediate concentration. To probe the regulation mechanisms for the dose-dependent response to pheromone, we selected functional genes that were associated with the response. First, we chose 79 genes that are either physically interact with Ste12 from *Saccharomyces* Genome Database (SGD) or are transcribed by Ste12 as revealed by a genome-wide localization study (p-value < 0.001) [2]. 64 genes relating to different functions including cell cycle, cell polarity and mating were selected manually from SGD by searching the descriptions of genes, creating a signature of 143 genes. We further added 52 transcriptional factors in budding yeast to reflect the changes in the transcriptional program [3], which brought the total number of genes in this study to 195 functional genes.

**Microfluidics device**

We used a high-throughput microfluidic chip in our fluorescence experiment, which allows a maximum of 96 parallel experiments across 2 different conditions (S1A Fig). Our chip was fabricated with PDMS (polydimethylsiloxane, RTV615, Momentive Performance Materials Inc.) using standard soft lithography technology. Each strain was loaded into an individual channel where the cells were trapped within an observation chamber approximately 4 μm high and 200 μm wide, which allowed a single layer growth of yeast cells. The loading cells were confined by the ‘fence-like’ structure by the side of the observation chamber to prevent contamination between different channels. Every 48 channels share the same two inlets of the culture medium, and the fresh medium passes through the ‘fence-like’ structure to reach the yeast cells. Before the microfluidic experiment, the chip was degassed for 15 min to assist the cell loading.

**Device operation and microscopy**

The culture medium was loaded into injection syringes, and the inflow of the medium was actuated by a syringe pump under the control of a TS-1B Syringe Pump Controller (LongerPump, Inc, Hebei, China) to achieve a constant flow rate of 400 μl/h. For each set of experiments (including 48 parallel experiments), the culture medium with and without alpha-factor was injected through separate inlets. Once loaded into the microfluidic chip, the yeast cells were cultivated using fresh medium for approximately 1 h before pheromone stimulation. Waste liquid was carried out of the chamber through loading wells and was absorbed by filter paper upon exiting the chamber throughout the experiment. Phase contrast and fluorescence images of yeast cells were generated via a Nikon Ti-E microscope in combination with NIS-Elements software. We selected two fields of view manually for each cell trap, contributing to 96 observation positions for 48 strains. The images were obtained every 5 min for each position for approximately 10 hours. A cell culture incubator around the microscope was used to maintain a temperature of 30℃.

**Measurement of protein abundance**

We modified an image processing pipeline in MATLAB to automatically segment cells and calculate GFP concentration from the obtained image series. Phase contrast images, in which the cell walls are brighter than the background, were used for cell segmentation. The images were first cropped to remove the chamber boundaries that were occasionally included in the field of view. A user-defined threshold was set for each channel to outline the boundary of the cells, resulting in a discrete map in which all the pixels whose value was greater than the threshold were marked. The program then found all of the connected components in the image and filled holes in the identified objects to generate masks for the cell areas. Polarization of yeast cells may produce breakpoints in the image of the cell boundary, which leads to incomplete masks. To overcome this problem, a dynamic directional gradient vector flow (DDGVF) algorithm was integrated into the pipeline to further refine the cell masks by incorporating directional gradient information and eliminating the negative curvature of the identified objects [4]. To exclude contaminations in the image, we removed all objects that were smaller than 50, or larger than 500 pixels in size.

The GFP concentration was calculated by processing the fluorescence images. Values of the pixels outside the segmented cells were used to calculate the background fluorescence. We fitted a 5^th^ polynomial surface to approximate the background fluorescence in the cell areas, whose value was subtracted from each pixel. The fluorescence value for each cell was generated by averaging the pixel values in the corresponding segment. The mean concentration of protein at a time point is the averaged fluorescence intensity of all of the cells in the field of view. We also developed a batch mode for the program to automatically process the image series from different channels.

**Measurement of growth rate**

Growth rate (cell mass accumulation rate) was calculated using a separate MATLAB script. Cell segmentation was obtained by a similar procedure as in the calculation of the GFP concentration. Increase in cell mass (dM) was generated by summing the difference between the two successive mask images. However, image shifting and imperfect cell segmentation could result in an over-estimation of the cell growth. To detect the image shift between successive images, a 2-D correlation coefficient was calculated for two successive images. For images with a correlation less than 0.8, we aligned them by finding the shift in two dimensions that maximized the correlation. When we attained the difference image of the two masks. We also excluded all objects that were larger than 100 pixels in size, which may result from the appearance (or disappearance) of a single cell. The exponential cell growth rate was generated by dividing dM with the sum of the cell areas in the former mask (M). The obtained growth rate was then smoothed and normalized to have the mean value of $7.7\times10^{-3}$/min for the first 5 time points, which is equivalent to a division time of 90 min.

**Estimation of protein synthesis rate**

We developed a kinetic model of protein dynamic change:

$$\frac{dP(t)}{dt}=\alpha(t)-(d+\gamma(t))P(t)$$

where *P*(*t*) is the protein concentration at time t, $\alpha(t)$ represents the protein synthesis rate and $\gamma(t)$ represents protein dilution rate, which is equivalent to the exponential cell mass accumulation rate. *d* is the protein degradation rate estimated from a genome-wide measurement of protein half-lives [5]. Because we had the time-resolved measurement of *P*(*t*) and $\gamma(t)$, it is possible to rewrite the equation to a discretized form and directly solve the protein synthesis rate. Profiles of the protein concentration and cell growth rate were smoothed at first to avoid undue amplification of noise. We also introduced an upper-limit (0.017) for *d* in the simulation, which corresponds to a half-life of 40 min. The estimated protein synthesis rate was further normalized by the initial value to allow for comparison between different genes.

**Protein synthesis decoupling analysis (PSDA)**

We suspected that the per-gene regulations in the response mainly took place in the protein synthesis term. Our kinetic model for protein concentration was thus modified to the following form:

$$\frac{dP(t)}{dt}=R(t)S(t)-(d+\gamma(t))P(t)$$

in which *S*(*t*) is the global synthesis rate, which reflects the changes of cellular resources related to protein synthesis, and *R*(*t*) is the temporal regulation term of the protein. *S*(*t*) was estimated by averaging the protein synthesis rate of the control genes for shmooing, which have a <0.2-fold change in the transcriptome data [6], or of all the investigated genes for elongated cells (S2 Fig). Because the absolute protein abundance varies across the genome, it is necessary to rescale the normalized protein synthesis rate to account for the difference in basal expression. For a particular gene, protein abundance in the normal state (a mean value over 25 min before pheromone stimulation) was used to calculate the rescaling factor, which is equal to$(d+\gamma(0))P_{control}$. *R*(*t*) is assumed to take a pulse-like form with three parameters, $R_{start}$,$R_{end}$ and $R_{FC}$, representing starting and ending time of regulation and the fold change, respectively, which captures the main characteristic of gene regulation [7, 8] and transforms the problem of regulation identification to a parameter estimation problem.

We adopted an efficient parameter optimization algorithm named differential stimulated annealing (DSA) to estimate the regulation parameters for each protein [9]. The calculation was based on a customized MATLAB version of the algorithm. In each round of parameter evaluation, we simulated the dynamics of protein concentrations and set the value of the objective function to the sum of squared errors between the simulated and observed trajectory. The calculation was terminated if either of the following two conditions is satisfied: i) the value of the objective function is less than or equal to the temporal variation of the protein abundance, which is the standard derivation of the difference between the original and smoothed time series data, or ii) the maximum number of objective function evaluations, i.e., 2000 times, is exceeded. The fold change of regulation$R_{FC}$was constrained to the range of [0.2, 20] and we also assumed that $R_{end}-R_{start}\geq50\min$in the parameter estimation.

**Cluster analysis and discretization**

Genes with a net fold change > 0.2 were selected for further analysis, resulting 141 functional genes for shmooing cells and 171 genes for elongated cells. The regulation patterns of 141 genes in shmooing cells were clustered using k-means clustering and hierarchical clustering, in which the hierarchical tree was generated using a Euclidean distance metric. Because hierarchical clustering with a cutoff would produce clusters that are either too large or too small, we chose k-means clustering with a cluster number of 6 to generate more balanced clusters and identify major regulation modes across the genes (S5A Fig). To obtain discrete trajectory for the system, a threshold model was applied to calculate the activation/inhibition time of the clusters. The time point when 50% of genes were activated (or inhibited) was assigned as the activation (inhibition) time, and two neighboring states were combined together if they were identical, leading to a trajectory of 11 steps (S2 Table). A threshold of 30% and 70% was also used to access the robustness of the results (S5B Fig). We found the relative order of activation/inhibition events in the discrete trajectory is robust against variations in the threshold. A threshold of 30% reproduces 95% of the relative sequence of events, and a threshold of 70% reproduces 87%.

We also conducted a further analysis based on the reviewer’s suggestion. C6 in the original manuscript (the 4^th^ cluster from top to bottom in S5 Fig) was divided into 3 new clusters (S6 Fig). The new time trajectory for the 8-node network includes 3 new states. We generated 32 minimal networks underlying this trajectory as shown in the C panel. We found all of the newly identified edges (green ones) direct to the new clusters and the general topology of the original network is unchanged. Our result indicates that the reconstructed network is robust to the clustering choices.

**Boolean network model**

In the Boolean network model, each node represents a biological species. We use$S_{i}(t)\in\left\{0,1\right\}$ to denote a state of node i at time t. Regulation from node j to node i is represented by the coefficient$a_{ji}$, which is positive for activation and negative for inhibition. As the Boolean trajectory is nearly unchanged when we increase the weight of repression [10], a dominant inhibition form of regulation was used in our simulation, in which$\left|a_{inh}\right|$≫$\left|a_{act}\right|$except for self-inhibitions. Thus node i is inhibited if at least one of its repressors is activated, regardless of all of the activation terms. State of nodes in the network are updated synchronously via the following rule:

$$\left\{\begin{matrix} \text{S}_{i}(\mathrm{t}+1)=\theta(\sum_{j} S_{j}(\mathrm{t})\mathrm{a}_{ji}),\sum_{j} S_{j}(\mathrm{t})\mathrm{a}_{ji}\neq0 \\ S_{i}(\mathrm{t}+1)=\mathrm{S}_{i}(\mathrm{t}),\sum_{j} S_{j}(\mathrm{t})\mathrm{a}_{ji}=0 \end{matrix}\right.$$

where$\theta$is the Heaviside step function as follows: $\theta(x)=1$when$x>0$and$\theta(\mathrm{x})=0$when$x<0$. From a given initial state, the state of the system is updated until it reaches a steady state known as an attractor. For each attractor, the number of the states that evolve to it is termed as its basin size.

**Boolean Network Reverse Engineering**

In the reverse engineering process, we tried to derive network structures from the observed trajectory. For the Boolean network model with dominant inhibition terms, the constraints on network topology can be written in logic expressions [11, 12]. The logic variable $\text{r}_{ji}$ is used to denote the inhibitory regulation from node j to node i. Similarly, $g_{ji}$ is used to represent the positive regulation, with$r_{ij}\cdot g_{ij}=0$. For a given trajectory, one can obtain the logic constraint of regulations on node i through the following logic expression,

$$s_{i}(t+1)=\left(\sum_{j\neq i} (s_{j}(t)\cdot g_{ji})+s_{i}(t)\cdot\overline{r_{ii}}+\overline{s_{i}(t)}\cdot g_{ii}\right)\cdot\prod_{j\neq i} (\overline{s_{j}(t)\cdot r_{ji}})$$

The OR gate is represented by ‘+’ and ‘∑’, while inhibition terms are combined by the AND gate, which is represented by ‘.’ and ‘∏’. The bar in the equation denotes the NOT gate. Logic expressions for each node at different time steps are combined together to obtain the final constraint of a trajectory, which is further used to generate all of the possible networks. For our 6-node system in the shmooing response, $4.1\times10^{8}$ possible networks are capable of producing the discrete trajectory obtained with a threshold of 50%.

**Further constraint on the network topology**

It has been proposed that the minimal network is the functional backbone of the biological regulatory network [10]. Previous work also provides evidence that biological networks might prefer to use minimal networks to fulfill their functions [12]. We here focused on the network topologies that were responsible for the observed dynamics and, hence, used the same assumption. To apply the minimal network constraint in our approach, we enumerated all possible regulations of each node and obtained the ones with the fewest edges. The regulations for each node were then combined together, leading to the identification of 96 minimal networks (S3 Table). Prior knowledge of the mating response pathway was also incorporated in the reverse engineering process to narrow the solution space. We suspected that cluster 3, which contains MAPK, Fus3 and Kss1, was directly activated by the input signal and cluster 5, which contains typical mating genes, such as Fus1 and Far1, should be stimulated by genes in cluster 3. This information reduced the number of minimal networks to 8. To generate the regulatory network in elongated cells, we eliminated all of the edges that are inconsistent with the dynamic changes of six clusters, including the activation edges of cluster 3 and cluster 6. Self-activation of cluster 1 is also included in the network to account for its activation (S7A Fig).

References

1. Ghaemmaghami S, Huh W-K, Bower K, Howson RW, Belle A, Dephoure N, et al. Global analysis of protein expression in yeast. Nature. 2003;425(6959):737-41.
2. Lee TI, Rinaldi NJ, Robert F, Odom DT, Bar-Joseph Z, Gerber GK, et al. Transcriptional Regulatory Networks in Saccharomyces cerevisiae. Science. 2002;298(5594):799-804.
3. Teixeira MC, Monteiro PT, Guerreiro JF, Goncalves JP, Mira NP, dos Santos SC, et al. The YEASTRACT database: an upgraded information system for the analysis of gene and genomic transcription regulation in Saccharomyces cerevisiae. Nucleic Acids Res. 2014;42(Database issue):D161-6.
4. Cheng J, Foo SW. Dynamic directional gradient vector flow for snakes. IEEE Trans Image Process. 2006;15(6):1563-71.
5. Belle A, Tanay A, Bitincka L, Shamir R, O'Shea EK. Quantification of protein half-lives in the budding yeast proteome. *Proc Natl Acad Sci U S A* 2006;103(35):13004-9.
6. Roberts CJ, Nelson B, Marton MJ, Stoughton R, Meyer MR, Bennett HA, et al. Signaling and circuitry of multiple MAPK pathways revealed by a matrix of global gene expression profiles. Science. 2000;287(5454):873-80.
7. Lee MV, Topper SE, Hubler SL, Hose J, Wenger CD, Coon JJ, et al. A dynamic model of proteome changes reveals new roles for transcript alteration in yeast. Mol Syst Biol. 2011;7:514.
8. Jovanovic M, Rooney MS, Mertins P, Przybylski D, Chevrier N, Satija R, et al. Immunogenetics. Dynamic profiling of the protein life cycle in response to pathogens. Science. 2015;347(6226):1259038.
9. Dai Z, Lai L. Differential simulated annealing: a robust and efficient global optimization algorithm for parameter estimation of biological networks. Mol Biosyst. 2014;10(6):1385-92.
10. Wang GY, Du CH, Chen H, Simha R, Rong YW, Xiao Y, et al. Process-based network decomposition reveals backbone motif structure. *Proc Natl Acad Sci U S A* 2010;107(23):10478-83.
11. Zhang X, Shao B, Wu Y, Qi O. A Reverse Engineering Approach to Optimize Experiments for the Construction of Biological Regulatory Networks. PLoS ONE. 2013;8(9):e75931.
12. Shao B, Wu J, Tian B, Ouyang Q. Minimum network constraint on reverse engineering to develop biological regulatory networks. J Theor Biol. 2015;380:9-15.
