# Supplementary material for: Reconstructing the regulatory circuit of cell fate determination in yeast mating response
Source: PLoS Comput Biol. 2017 Jul 24;13(7):e1005671. doi: 10.1371/journal.pcbi.1005671 (PMC5546706; doi:10.1371/journal.pcbi.1005671)
Supplement: S1 Table — (DOCX) [file pcbi.1005671.s002.docx]

**S1 Table. Percentage of gene ontology terms in the clusters.**

| GOID | Gene Ontology (GO) term | All (141) | C1 (14) | C2 (21) | C3 (11) | C4 (21) | C5 (31) | C6 (43) |
| --- | --- | --- | --- | --- | --- | --- | --- | --- |
| 6366 | transcription from RNA polymerase II promoter | 40.4% | 42.9% | 47.6% | 45.5% | 42.9% | 48.4% | 27.9% |
| 278 | mitotic cell cycle | 12.8% | 7.1% | 23.8% | 18.2% | 14.3% | 6.5% | 11.6% |
| 33043 | regulation of organelle organization | 11.3% | 21.4% | 19.0% | 9.1% | 0% | 9.7% | 11.6% |
| 6325 | chromatin organization | 19.1% | 28.6% | 19.0% | 9.1% | 14.3% | 22.6% | 18.6% |
| 42221 | response to chemical | 28.4% | 35.7% | 14.3% | 54.5% | 23.8% | 38.7% | 23.3% |
| 23052 | signaling | 14.2% | 28.6% | 14.3% | 27.3% | 14.3% | 12.9% | 9.3% |
| 746 | conjugation | 16.3% | 14.3% | 14.3% | 9.1% | 19.0% | 25.8% | 11.6% |
| 6354 | DNA-templated transcription, elongation | 7.1% | 7.1% | 14.3% | 0% | 0% | 0% | 11.6% |
| 7124 | pseudohyphal growth | 2.1% | 7.1% | 9.5% | 18.2% | 4.8% | 6.5% | 11.6% |

4 most enriched GO terms in each of the 6 clusters were selected. Annotations of the genes were mapped using Gene Ontology Slim Mapper in S. cerevisiae Genome Database (SGD). We underlined the cluster with largest probability for each GO term. Numbers of genes in each cluster are labeled in the bracket.
