# Supplementary material for: Reconstructing the regulatory circuit of cell fate determination in yeast mating response
Source: PLoS Comput Biol. 2017 Jul 24;13(7):e1005671. doi: 10.1371/journal.pcbi.1005671 (PMC5546706; doi:10.1371/journal.pcbi.1005671)
Supplement: S2 Table — (DOCX) [file pcbi.1005671.s003.docx]

**S2 Table. Time trajectory of the system under high concentration of pheromone.**

| Time(min) | C1 | C2 | C3 | C4 | C5 | C6 | Input |
| --- | --- | --- | --- | --- | --- | --- | --- |
| normal | 1 | 1 | 0 | 0 | 0 | 0 | 0 |
| T = 0 | 1 | 1 | 0 | 0 | 0 | 0 | 1 |
| T = 1* | 1 | 1 | 1 | 0 | 0 | 0 | 1 |
| T = 9 | 0 | 1 | 1 | 0 | 0 | 0 | 1 |
| T = 43 | 0 | 1 | 1 | 1 | 0 | 0 | 1 |
| T = 51 | 0 | 1 | 0 | 1 | 0 | 0 | 1 |
| T = 57 | 0 | 1 | 0 | 1 | 1 | 0 | 1 |
| T = 115 | 0 | 0 | 0 | 1 | 1 | 0 | 1 |
| T = 152 | 0 | 0 | 0 | 1 | 1 | 1 | 1 |
| T = 172 | 1 | 0 | 0 | 1 | 1 | 1 | 1 |
| T = 202 | 1 | 0 | 0 | 0 | 1 | 1 | 1 |
| T = 350** | 1 | 0 | 0 | 0 | 1 | 1 | 1 |

*T = 1 is added to the trajectory manually, in which cluster 3 is activated.

**State at T = 350 corresponds to the attractor of the system.

The time trajectory was generated by using a threshold of 50% from identified per-gene regulations in shmooing cells. C1-C6 represents cluster 1-6, respectively.
