# Supplementary material for: Reconstructing the regulatory circuit of cell fate determination in yeast mating response
Source: PLoS Comput Biol. 2017 Jul 24;13(7):e1005671. doi: 10.1371/journal.pcbi.1005671 (PMC5546706; doi:10.1371/journal.pcbi.1005671)
Supplement: S3 Table — (DOCX) [file pcbi.1005671.s004.docx]

**S3 Table. The rigid and interchangeable edges of all the nodes in shmoo formation.**

| Node | Rigid Edges | Interchangeable Edges | Number of Possible Regulations |
| --- | --- | --- | --- |
| C1 | *r*_31_, *g*_61_ | - | 1 |
| C2 | *r*_52_ | - | 123 |
| C3 | (*r*_43_, *g*_i3_) | (*r*_63_, *r*_53_) | 107 |
| C4 | *r*_14_ | (*g*_24_, *g*_44_, *g*_34_, *g*_i4_*) | 80 |
| C5 | *r*_35_ | (*g*_25_, *g*_45_*, *g*_i5_) | 3 |
| C6 | *r*_26_ | (*g*_66_, *g*_46_, *g*_56_, *g*_i6_) | 129 |

*interchangeable edges that are supported by literature.

The rigid edges and interchangeable edges with asterisks are presented in Fig 4. Reverse engineering of Boolean network model identified a total of $4.1\times10^{8}$possible networks, with 96 minimal networks. Rigid edges are the ones that exist in all possible networks, while different combinations of interchangeable edges result in different minimal networks.
