# Supplementary figures and images for: Reconstructing the regulatory circuit of cell fate determination in yeast mating response
Source: PLoS Comput Biol. 2017 Jul 24;13(7):e1005671. doi: 10.1371/journal.pcbi.1005671 (PMC5546706; doi:10.1371/journal.pcbi.1005671)

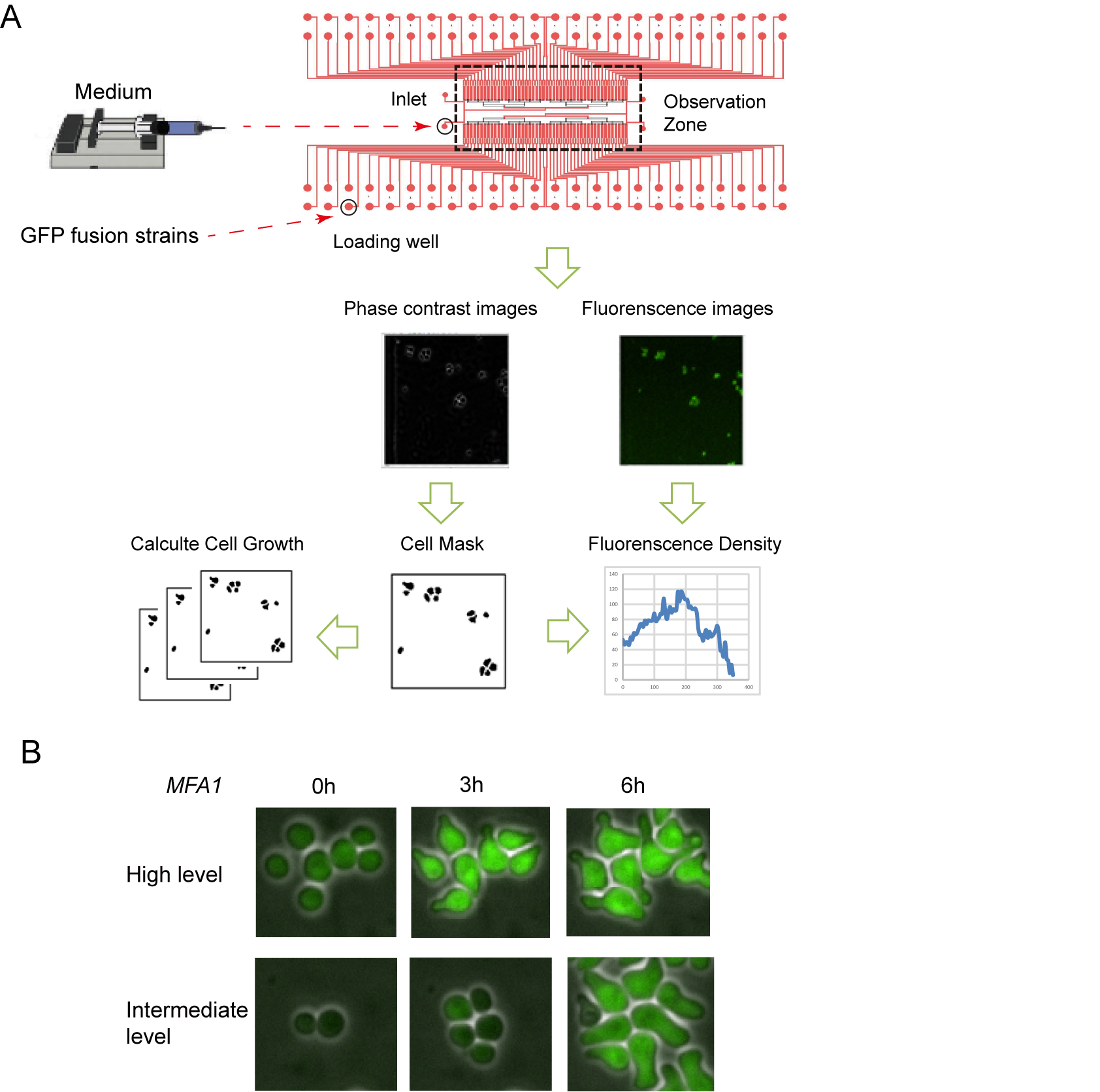

Supplement: S1 Fig — (A) The image processing pipeline. The microfluidic chip allows maximumly 96 parallel experiments. The observation chamber in each channel is about 4 μm high and 200 μm wide. Growth medium was loaded into the chip from injection syringes driven by pumps and GFP-tagged strains were loaded through the wells by the side. The fluorescence microscope scanned over the chip every 5 min to generate phase contrast and fluorescence images of the yeast cells. The phase contrast images were used in cell segmentation, which provide basis for measuring GFP concentration and estimation of cell growth rate. (B) Microfluidic experiment reproduces different phenotypes of yeast cells in mating response. When exposed to high level of alpha factor, the yeast cells formed small projections sequentially to search for mating partner in the surrounding area. Under a lower dose of pheromone, the cells arrested their cell cycles and began chemotrophic growth, usually resulting in a radial pattern of colony. (TIF) [file pcbi.1005671.s008.tif]

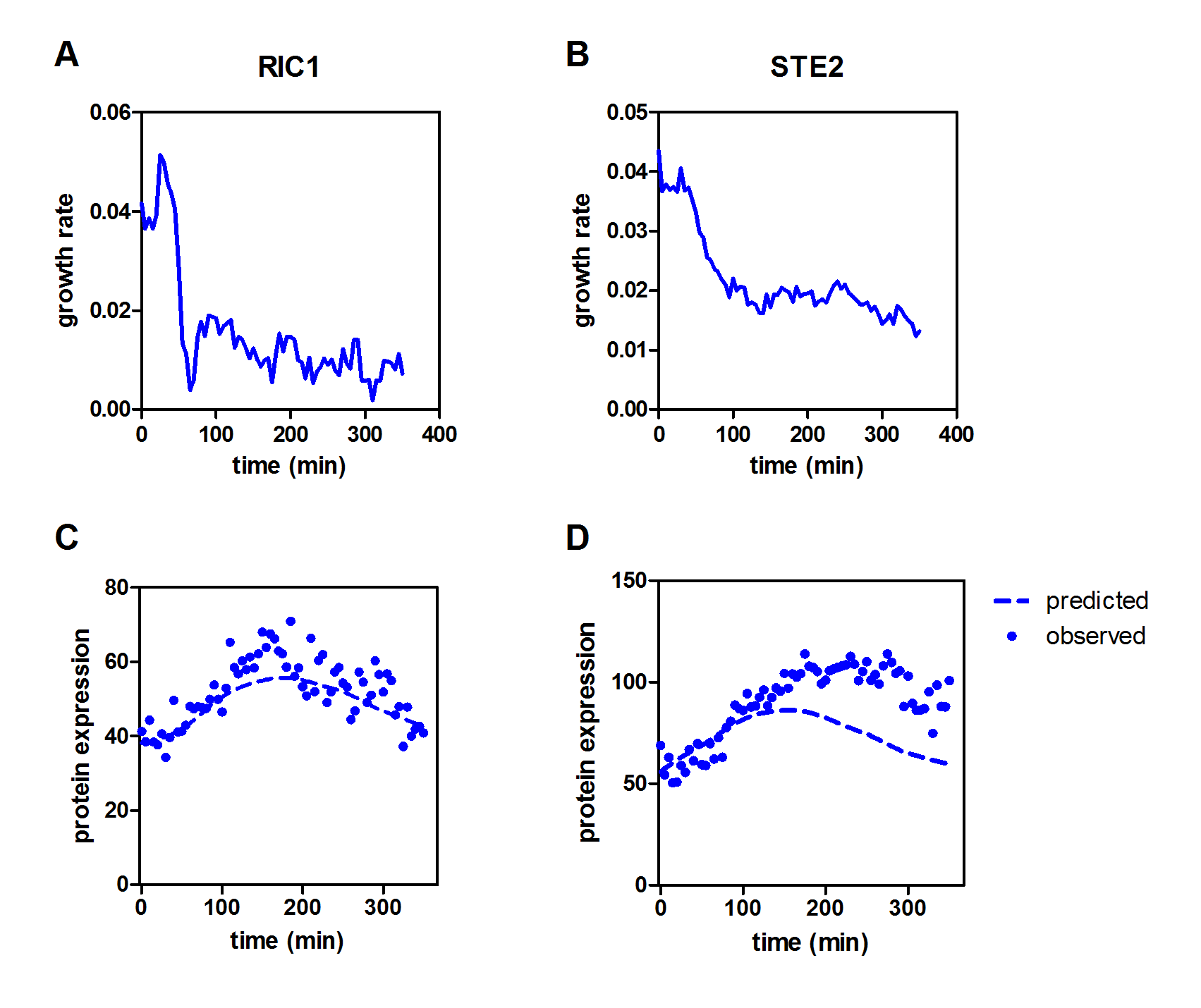

Supplement: S3 Fig — The measured growth rate of two different yeast strains in shmoo formation, in which GFP is tagged to RIC1 (A) and STE2 (B). GFP concentration for the strains (dot) and protein dynamic profiles predicted from global regulation (dashed line) are illustrated in (C, D) respectively. The discrepancy of measured and predicted profile reveals the time window and relative level of per-gene regulation, with STE2 activated in the latter phase and no significant regulation for RIC1. (TIF) [file pcbi.1005671.s010.tif]

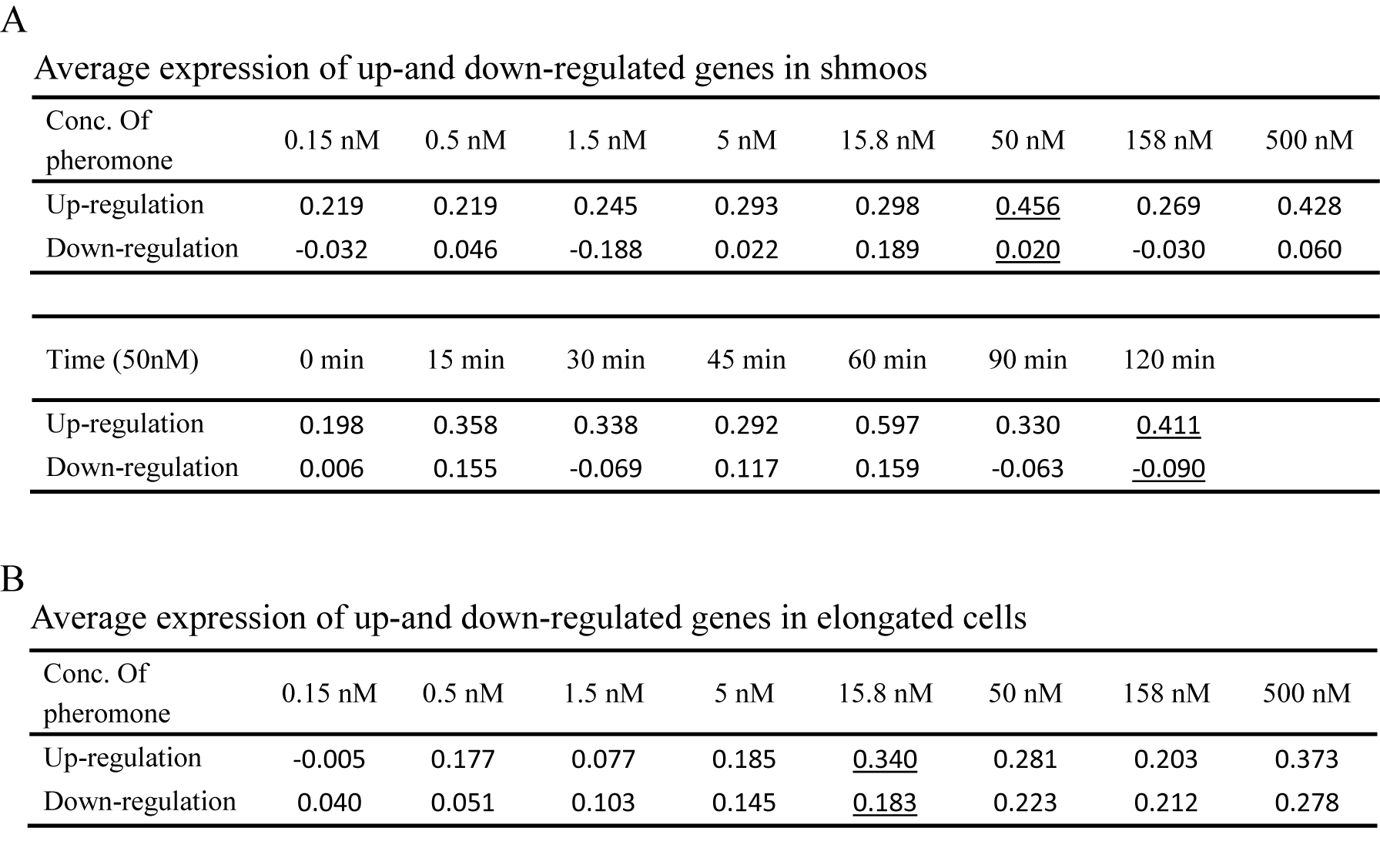

Supplement: S4 Fig — (A) Expression of up- and down-regulated genes in shmooing cells. Average expression value was generated by reprocessing data from [28], in which bar1 mutant cells were used. Data point with largest divergence between up- and down-regulated genes was underlined. (B) Expression of up- and down-regulated genes in elongated cells. (TIF) [file pcbi.1005671.s011.tif]

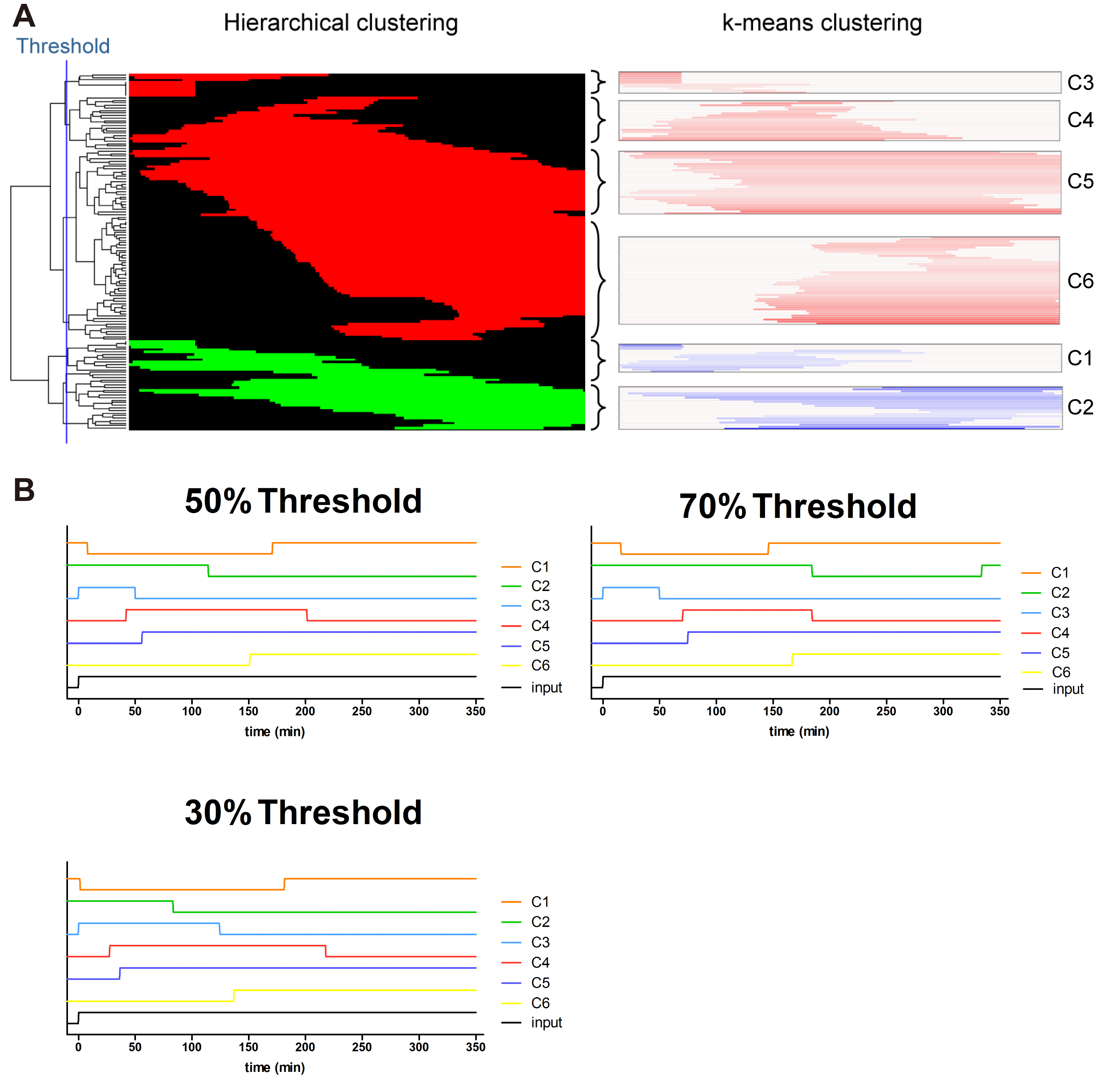

Supplement: S5 Fig — (A) Hierarchical clustering and k-means clustering of gene-specific regulations in shmooing. Temporal modes of gene regulation events of 141 genes in shmooing cells was clustered by two methods. By introducing a threshold in hierarchical clustering and aligning the corresponding clusters of two methods, we found that the clusters identified by k-means clustering either recapture, or rearrange the clusters from hierarchical clustering, thus providing a more balanced dividing of the genes. (B) Robustness of the threshold model in discretizing time trajectory. The discrete time trajectories were generated by using different thresholds, i.e., 50%, 30% and 70%. Different colors denote different clusters. The relative sequence of regulation events were conserved in respect to the variations in threshold. (TIF) [file pcbi.1005671.s012.tif]

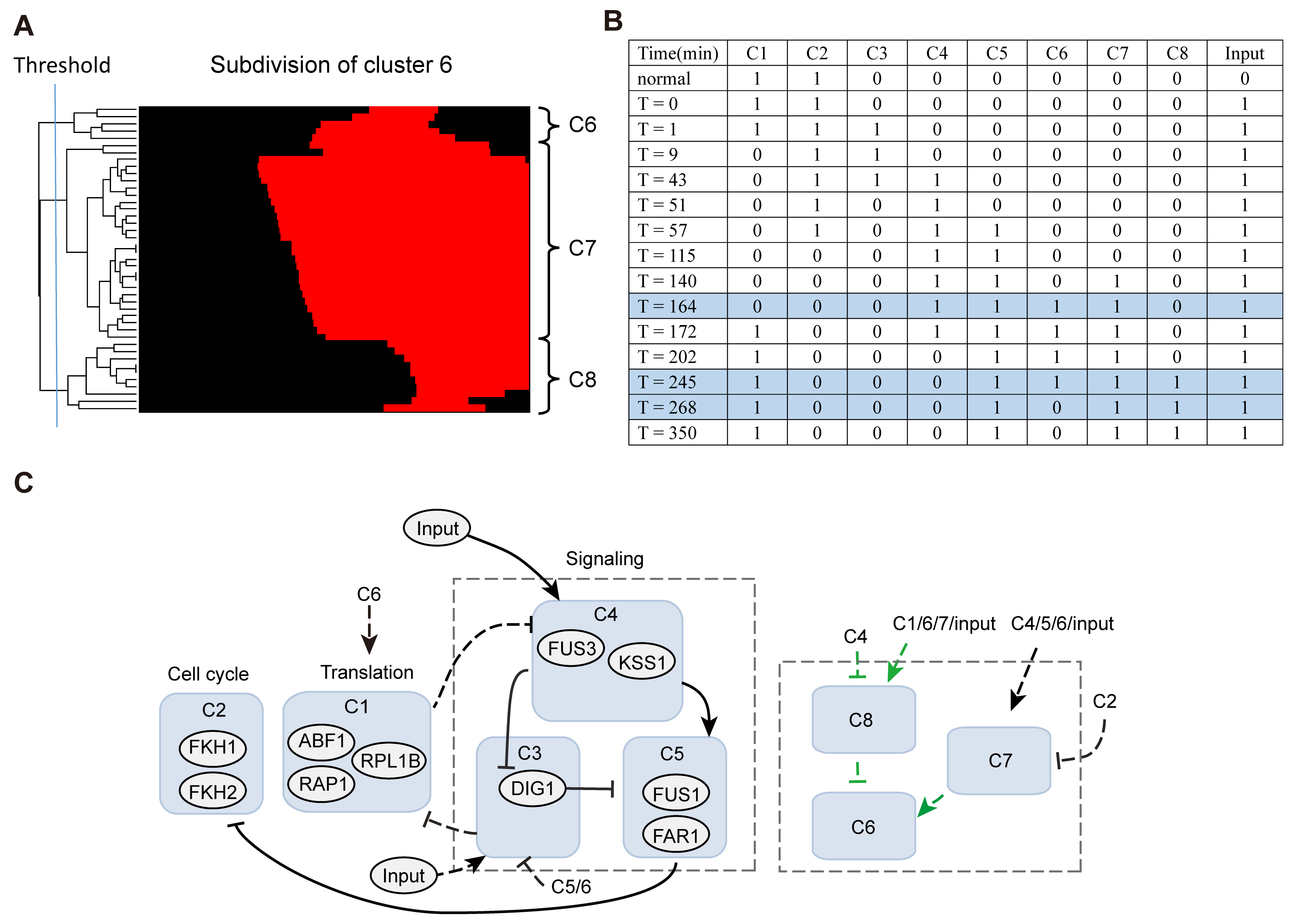

Supplement: S6 Fig — (A) Hierarchical clustering of C6 results in 3 sub-clusters with different expression program in latter phase of mating response. (B) Discrete time trajectory for the 8-node network. New states relative to S2 Table is shaded in blue. (C) The resulting 8-node network. Addition of 2 clusters leads to new edges (green) but doesn’t alter the topology of the original network. (TIF) [file pcbi.1005671.s013.tif]

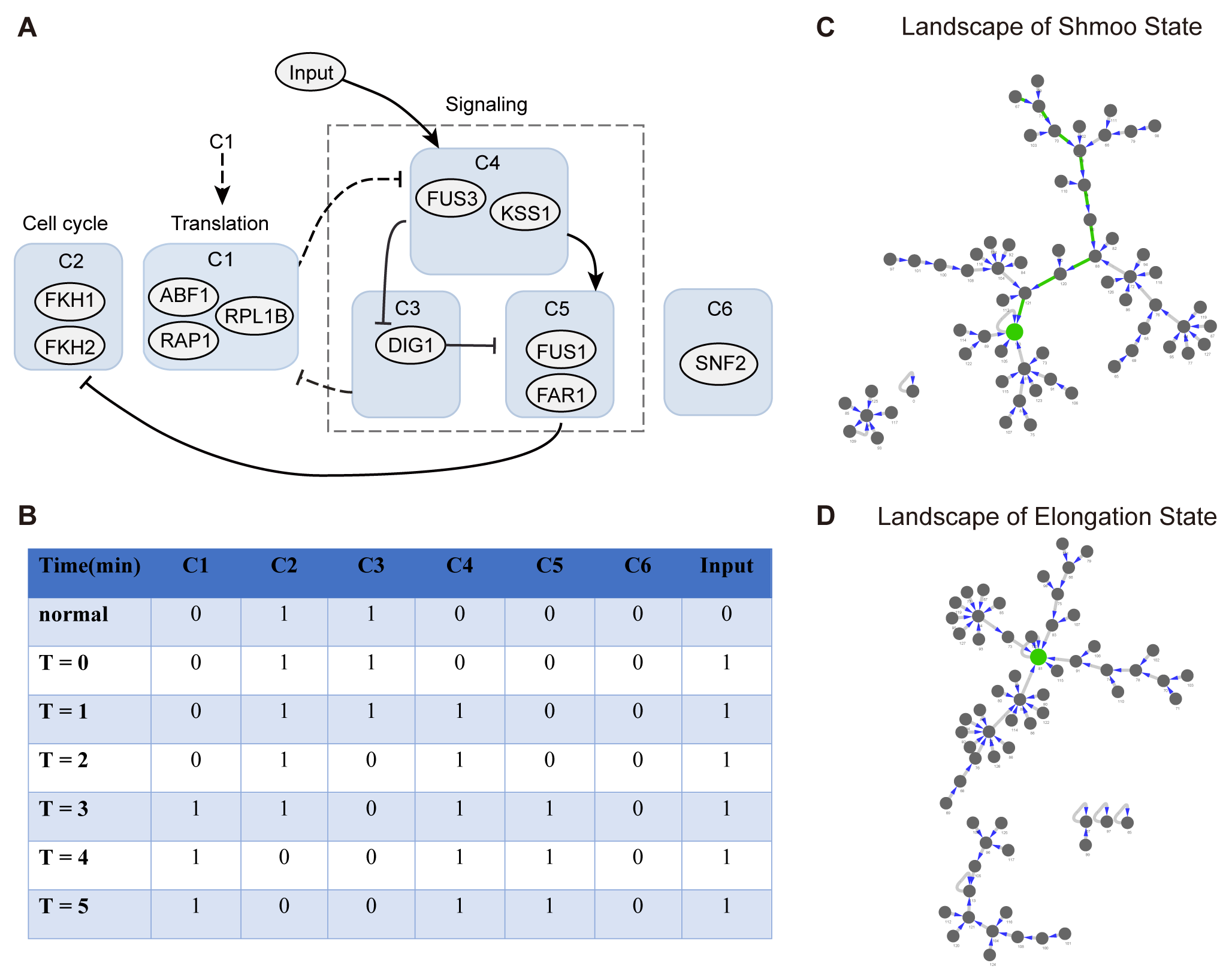

Supplement: S7 Fig — (A) Gene regulatory circuit for elongated cells. Solid and dashed edges are used to denote the canonical pheromone response pathway and novel regulations, respectively. Edges with bar-end, regulation of inhibition; edges with arrow-end, activation. (B) Time trajectory of the regulatory circuit. The trajectory was generated by using the regulatory network in elongated cells, in which 3 edges were altered in respect to the differential regulations of six clusters. The initial state was changed due to the different regulation modes of C1 and C3 in elongated cells. The time trajectory recapitulates the dynamic changes of different clusters in elongated growth, as shown in Fig 5A. (C) Attractor landscape for shmoo formation. For 6-node Boolean network, there’re 26 = 64 possible states (gray point). We selected a minimal network from Fig 4B to calculate Si(t+1) from a give state Si(t). Dynamic flow from one state to another is indicated by the directed arrow, with green arrows representing the biological trajectory in shmooing cells (S2 Table). Most of the states (gray points) converge to the biological state (1, 0, 0, 0, 1, 1, 1) (green point) of shmoo. Input value was set to 1 at all times. (D) Attractor landscape for elongated growth. Network from S7A Fig was used to update the state of network. We assume there’s a self-inhibition edge for cluster 6 to keep it in the inactivated state. Green point indicates the attractor in elongated growth, i. e. (1, 0, 0, 1, 1, 0, 1). (TIF) [file pcbi.1005671.s014.tif]

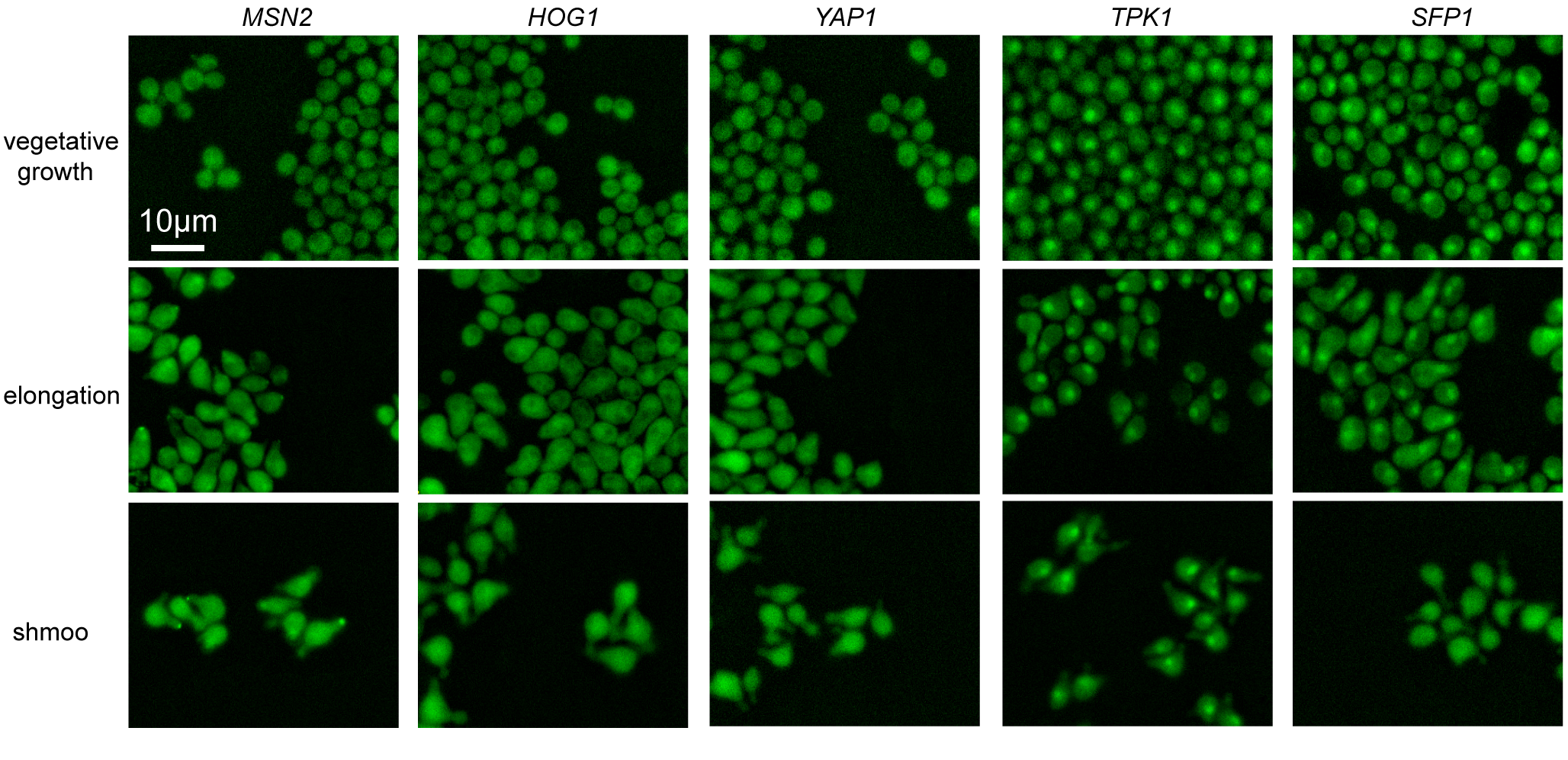

Supplement: S8 Fig — Images of the same live cells were captured after 4 hours of pheromone addition by fluorescence microscopy. The subtract background filter of ImageJ (US National Institutes of Health) was applied with a rolling ball radius of 30 pixels to remove background noise. (TIF) [file pcbi.1005671.s015.tif]
